# Geographic patterns in wildland fire exposures and county-level lung cancer mortality in the United States

**DOI:** 10.1186/s12942-025-00394-x

**Published:** 2025-04-11

**Authors:** Richard V. Remigio, Ian D. Buller, Michael S. Bogle, Maria E. Kamenetsky, Samantha Ammons, Jesse E. Bell, Jared A. Fisher, Neal D. Freedman, Rena R. Jones

**Affiliations:** 1 Occupational and Environmental Epidemiology Branch, Division of Cancer Epidemiology and Genetics, National Cancer Institute, 9609 Medical Center Drive, MSC 9771, Rockville, MD 20892-7991 USA; 2 DLH, LLC, Bethesda, MD USA; 3 Forest Service, United States Department of Agriculture, Salt Lake City, UT USA; 4 Department of Environmental, Agricultural, and Occupational Health, College of Public Health, University of Nebraska Medical Center, Omaha, NE USA; 5 Daugherty Water for Food Global Institute, University of Nebraska, Lincoln, NE USA; 6 School of Natural Resources, University of Nebraska, Lincoln, NE USA; 7 Tobacco Research Branch, Division of Cancer Control & Population Sciences, National Cancer Institute, Rockville, MD USA

**Keywords:** Wildland fires, Lung cancer mortality, Spatial analysis, Clustering, Smoking prevalence

## Abstract

**Background:**

Emissions from wildfire plumes are composed of modified biomass combustion by-products, including carcinogens. However, studies of the association between wildland fires (WF; includes wildfires, prescribed burns, and resource management fires) exposure and lung cancer are scant. We evaluated geographic patterns in these exposures and their association with lung cancer mortality (LCM) rates across the conterminous United States (US).

**Methods:**

We extracted data from the Monitoring Trends in Burn Severity program (1997–2003) and derived county-level exposure metrics: WF density by area, WF density by population, the ratio between total burned land area and county area, and the ratio between total burned land area by population. We obtained sex-specific, county-level LCM rates for 2016–2020 from the National Center for Health Statistics. Counties with fewer than 10 cases were suppressed. To account for cigarette smoking, we first modeled residual values from a Poisson regression between cigarette smoking prevalence and sex-specific, age-adjusted LCM rates. We then used Lee’s L statistic for bivariate spatial association to identify counties with statistically significant (*p* < 0.05) associations between WF exposures and these residuals. In a sensitivity analysis, we applied a false discovery rate correction to adjust for multiple comparisons.

**Results:**

We observed geographic variation in bivariate associations between large WFs and subsequent LCM rates across US counties while accounting for ever cigarette smoking prevalence. There were positive (high WF exposures and high LCM rate) clusters for males and females in counties within the mid-Appalachian region and Florida, and modest differences across WF metrics in the cluster patterns were observed across the Western US and Central regions. The most positive clusters were seen between WF density by area and LCM rates among women (*n* = 82 counties) and a similar geographic pattern among men (*n* = 75 counties). Similar patterns were observed for males and females in the western US, with clusters of high WF exposures and low LCM rates. After adjusting for multiple comparisons, a positive cluster pattern among both sexes persisted in Kentucky and Florida with area-based exposure metrics.

**Discussion:**

Our analysis identified counties outside the western US with wildfires associated with lung cancer mortality. Studies with individual-level exposure-response assessments are needed to evaluate this relationship further.

**Supplementary Information:**

The online version contains supplementary material available at 10.1186/s12942-025-00394-x.

## Introduction

Increases in wildfire occurrence over the last few decades have posed environmental health threats in the United States (US) and worldwide [1–3]. The National Interagency Fire Center reported in May 2024 that the year-to-date annual area burned has already exceeded the 10-year average of normal burned area in the US [4]. The magnitude and frequency of wildland fires (WF), which include wildfires, prescribed burns, and resource management fires, vary across the US [5]. From 1984 to 2021, approximately 12,463 large-scale WFs were recorded with an annual burned rate of over 485,738 acres per year [6]. Smoke plumes derived from WFs can last from a few days to multiple weeks. In addition to localized air quality impacts, plumes can travel distances downwind from their point of origin, and, consequently, affect ambient air quality over large regions [7–9]. 

Wildfires, a major class of WFs, have become more extensive and severe over time in the US [10, 11]. This trend raises concerns about smoke plumes reaching regions not typically exposed to WF-derived air pollution [12, 13] and associated health impacts [3, 14]. For example, in the summer of 2023, unprecedented long-range smoke plumes from Canadian wildfires reached the mid-Atlantic and northeastern US, contributing to dramatically worsened air quality. Asthma-associated emergency room visits were 17% higher than expected across the US on days with wildfire smoke during this event [15]. Similar studies focusing on short-term exposure periods found positive associations between ambient emissions of fine particulate matter (PM_2.5_, < 2.5 μm in diameter) derived from WFs and increased emergency department visits [16], hospital admissions [17], and mortality [18] related to cardiovascular, cerebrovascular, and respiratory outcomes. In 2023, the Fifth National Climate Assessment concluded *with very likely*,* high confidence* that wildfire emissions will increase in the future. As a result, wildfire emissions will likely continue to contribute to the risk of adverse health outcomes, including cardiovascular- and respiratory-related causes of morbidity and mortality [12]. 

WF smoke plumes include a mixture of modified biomass combustion by-products [9, 19]. These include known and potential carcinogens, including hexavalent chromium [20], polycyclic aromatic hydrocarbons (PAHs) [21], acrolein [22], acrylonitrile [23], benzene [24, 25], and formaldehyde [26]. The International Agency for Research on Cancer (IARC) recently classified occupational exposures common from firefighting, including wildfires, as carcinogenic to humans (Group 1). This determination was based on sufficient evidence for mesothelioma and bladder cancer in humans and strong evidence from mechanistic studies of genotoxicity and oxidative stress [27]. Exposure sampling studies among wildland firefighters handling prescribed fires have detected and measured harmful carcinogenic compounds in breathable air (ambient and personal) and on the skin despite the use of personal protection equipment [27]. In addition, multiple studies have reported the detection of biomarkers (e.g., volatile organic compounds and PAHs) of exposure from urine, blood, saliva, and breath samples, suggesting internal exposures among firefighters [27–29]. 

Despite biological plausibility and evidence of increased risk in worker populations, potential cancer risks associated with WF exposure in the general population have not been well studied. A registry-based cohort study in Canada found positive associations between residential proximity to wildfire exposures and the risk of lung and brain cancers [30]. A study in Brazil found increased mortality from multiple malignancies, including nasopharyngeal, stomach, skin, and reproductive organ sites, associated with wildfire-related PM_2.5_ compared to non-wildfire-related PM_2.5_ [31]. Investigation of these relationships in the US remains limited, except for a recent study that found reduced survival following wildfire exposures during the first year of recovery following lung cancer surgery [32]. 

This ecologic study evaluated geographic patterns in associations between WF exposures and lung cancer mortality (LCM) across the conterminous United States (CONUS). We focused on large wildland fires occurring within CONUS counties from 1997 to 2003 and lung cancer deaths 13 to 23 years later (2016–2020).

## Methods

### Wildland fire data and exposure assessment

We obtained information on WF events from the Monitoring Trends in Burn Severity (MTBS) database, cataloged by the US Geological Survey Center for Earth Resources Observation and Science and the US Department of Agriculture Forest Service (USFS) Geospatial Technology and Applications Center [33]. The MTBS data includes information on fire occurrences and estimated burned areas. These events include larger fire sizes spanning 1,000 acres or more in the western US and 500 acres or more in the eastern US [34]. This definition of large fires represents the majority (~ 95%) of annual burned area across the US [35]. The initial fire locations are geocoded and recorded based on the ignition date (day, month, and year).

We created multiple metrics to reflect the county-level burden of wildland fires between 1997 and 2003; we selected this exposure period to align with available data on the county-level smoking prevalence (discussed below). WF density by county area (Eq. 1) was calculated by dividing the total number of wildland fires ($\:N$) within county $\:i$ by the land area ($\:A$) of county $\:i$:

1 $$\:{d}_{i}=\frac{{N}_{i}}{{A}_{i}}$$

The area burned ratio (Eq. 2) was calculated by dividing the sum of total area burned ($\:B$) by WF event $\:j$ in county $\:i$ by the land area ($\:A$) of county $\:i$:

2 $$\:{b}_{i}=\frac{\sum\:_{j=1}^{n}{B}_{ij}}{{A}_{i}}$$

Because counties are also not uniform in population size, we created separate metrics that accounted for population density using data from the 2000 US decennial census. WF density by county population ($\:{\delta\:}_{i}$) was calculated by dividing the number of wildland fires (*N*) within county *i* by the population of county *i* (*p*_*i*_) multiplied by 100,000 persons (Eq. 3), and the area burned ratio by population ($\:{\beta\:}_{i}$) was calculated by the sum of $\:{B}_{ij}$ in county *i* divided by county *i* population, *p*_*i*_, multiplied by 100,000 persons (Eq. 4).

3 $$\:{\delta\:}_{i}=\frac{{N}_{i}}{{p}_{i}}\times\:\text{100,000}$$

4 $$\:{\beta\:}_{i}=\frac{\sum\:_{j=1}^{n}{B}_{ij}}{{p}_{i}}\:\times\:\text{100,000}$$

### Cigarette smoking prevalences

Prevalence of ever- and current-smoking at the county level were obtained from the National Cancer Institute’s Small Area Estimates for Cancer-Related Measures from 1997 to 2003. These sex-specific, self-reported estimates were derived from the Behavioral Risk Factor Surveillance System and the National Health Interview Study. Ever-smokers are defined as individuals over 18 years of age reporting having smoked at least 100 cigarettes in their lifetime by the time of the interview. Current smokers included ever cigarette smokers who also reported smoking cigarettes every day or some days.

### Lung cancer mortality ascertainment

We extracted LCM (ICD-10 code C34: malignant neoplasm of lung and bronchus; LCM) data from the National Vital Statistics System, including deaths among adults 20 years or older. County-level LCM counts and rates between 2016 and 2020 by sex were summed and then calculated using SEER*Stat version 8.3.9 [36]. This time period included the most updated available LCM data and represented a biologically plausible latency of more than 13 years with respect to the exposure period of 1997–2003. Age-adjusted LCM rates were standardized to the 2000 US population and expressed as deaths per 100,000 persons. Counties with fewer than 10 LCM deaths across the 5-year period were suppressed from our analysis, consistent with the National Center for Health Statistics reporting guidelines. This restriction yielded 2,577 and 2,700 counties for analyses of females and males, respectively (Table S-1).

### Statistical analyses

We described WF events and LCM rates across the conterminous US and by USFS Regions (S-1). Because some counties intersected more than one USFS region, we generated area-weighted summary statistics in each region.

**Fig. 1:**
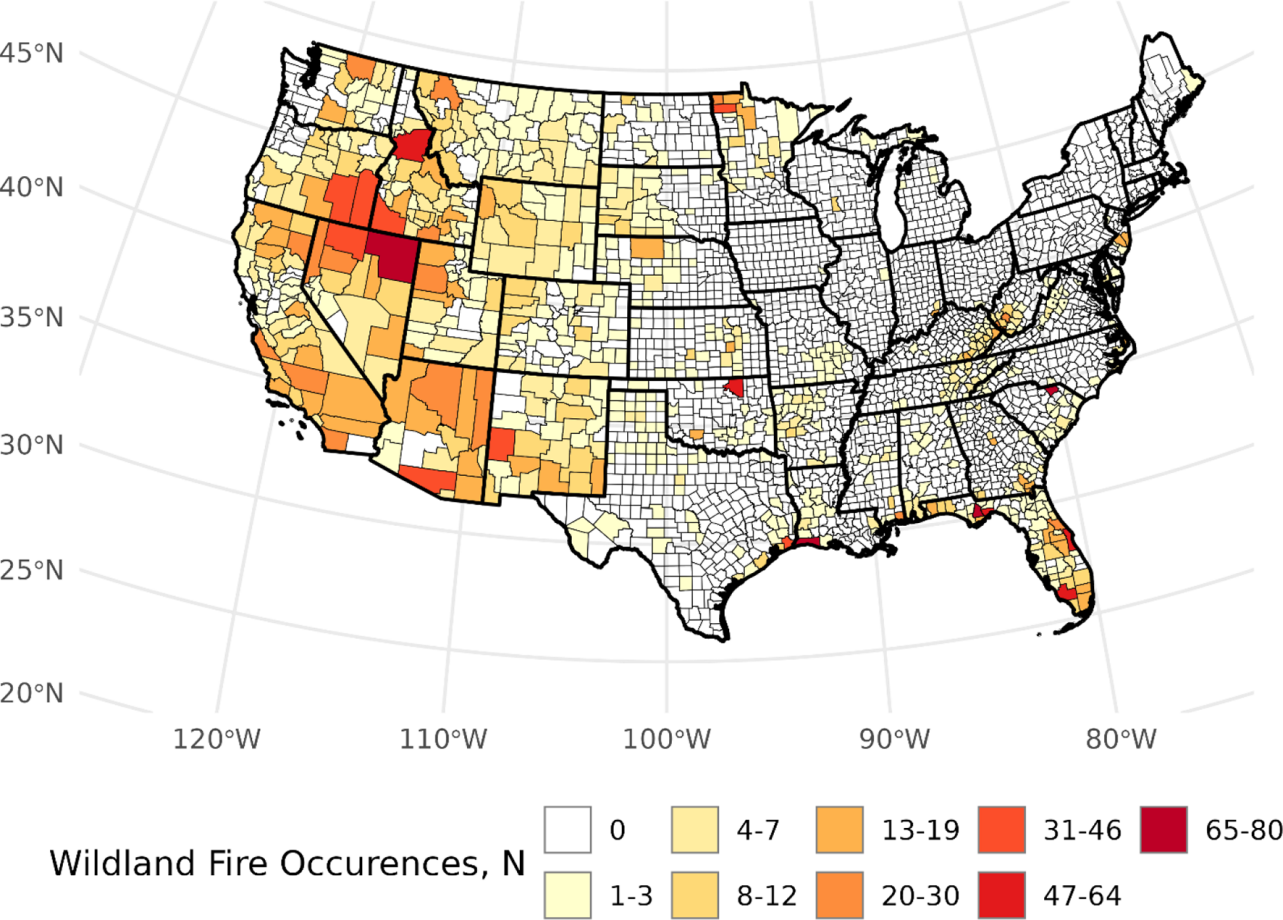
Distribution of wildland fire occurrences across conterminous United States counties, 1997–2003

We applied a two-stage analysis approach. To account for cigarette smoking prevalence, we extracted deviance residual values from a Poisson regression fit between smoking prevalence and sex-specific, age-adjusted lung cancer mortality rates (Stage 1). We generated these residuals for Poisson models of ever and current-smoking prevalence separately. We then modeled the bivariate spatial association of these residuals with WF metrics using the Lee’s L statistic [37]. The method integrates Pearson’s *r* and Moran’s *I* between exposure and outcome variables [38] by incorporating non-spatial correlations within the county and correlations of spatially lagged values, respectively. A Queen’s case adjacency matrix was used to assess spatial neighbors. Bivariate clusters between each WF prevalence metric and sex-specific LCM rates yielded four potential combinations of cluster type correlations: high WF prevalence and high LCM rate (high-high), low WF prevalence and high LCM rate (low-high), high WF prevalence and low LCM rate (high-low), and low WF prevalence and low LCM rate (low-low). The empirical *p*-values for spatial associations between each WF metric and LCM across counties were estimated by conducting 100,000 random permutations for each analysis. In a sensitivity analysis, we also applied a false discovery rate (FDR) correction [39] to account for multiple comparisons and adopted an initial alpha level of 0.05 to determine appropriate critical *p*-values. All analyses were performed using R (version 4.0.5; R Development Core Team).

## Results

From 1997 to 2003, 4,156 large WFs were cataloged, with the most occurrences within the Southern USFS region (*n* = 1,452; Table 1). Similarly, the Southern region had the highest county-level fire densities (14.0 per 1,000 km squared), whereas the Northern Region had the lowest (2.5 per 1,000 km squared). More granularly, we observed that counties with the highest county-level fire occurrences were found in South Carolina, Louisiana, Florida, Nevada, and Idaho. (Fig. 1). Over 24.7 million acres of burned area across CONUS were estimated based on the MTBS database. The highest and lowest USFS Region-level burned area ratios were observed in the Intermountain (*b* = 0.19) and Eastern regions (*b* = 0.03), respectively. Individual counties in New Mexico, Oregon, Florida, Kansas, and Colorado had among the highest burned area ratios *b* > 0.25). When considering population-based metrics at the USFS regional level, the Northern region exhibited the highest rate of normalized wildland fire events (7.67 per 100,000 persons). The Pacific Southwest Region had the lowest rate (0.26 per 100,000 persons). New Mexico, Florida, Louisiana, Nevada, and Montana had counties with the highest population-based WF density (*δ* > 1000). The Intermountain USFS region exhibited the highest population-normalized area burned. Similar to population-normalized fire density, western states, including Kansas, Wyoming, and Idaho, had the highest population-normalized area burned ratio (*β* > 5,000,000 acres per 100,000 persons). All 48 lower states and the District of Columbia had at least one county with no recorded WF occurrence between 1997 and 2003, with Texas (*n* = 199) and Georgia (*n* = 135) possessing the most counties with no large wildland fire exposures (Fig. 1). Large-scale fire occurrences across major portions of New England, mid-Atlantic, and Midwestern states were also not recorded.

**Table 1:** Summary of large wildland fires [1] across the conterminous United States (US), 1997–2003, overall and by US forest service regions [2]

Characteristic	Overall^2^	Eastern	Intermountain	Northern	Pacific Northwest	Pacific Southwest	Rocky Mountain	Southern	Southwestern
**Number of wildland fires**	4,156	351	646	282	289	376	370	1,452	390
**Fire density by area**^**3**^**(*n* / 1**,**000 km** **squared)**	4.82	3.91	4.70	2.47	2.54	3.90	3.28	13.96	4.19
**Fire density by population**^**4**^**(*n* / 100**,**000 persons)**	1.49	1.08	7.55	7.67	1.51	0.26	3.93	3.17	2.65
**Area burned (millions of acres)**	24.72	0.63	6.38	2.69	3.04	3.12	2.24	3.95	2.68
**Burned area to county area ratio** ^**5**^	0.12	0.03	0.19	0.10	0.11	0.16	0.08	0.17	0.12
**Burned area ratio by population**^**6**^**(Burned Area / 100**,**000 persons)**	8,843	1,921	74,590	73,227	15,827	2,142	23,766	8,620	18,176

^1^ Spanning 1,000 acres or more in the western US and 500 acres or more in the eastern US), per the Monitoring Trends in Burn Severity (MTBS) database

^2^ Area-weighted estimates

^3^ Number of fires / total land area (per 1,000 km squared)

^4^ Number of fires /population (per 100,000 persons)

^5^ Land area burned / total land area

^6^ Land area burned / population (per 100,000 persons)

**Fig. 2:**
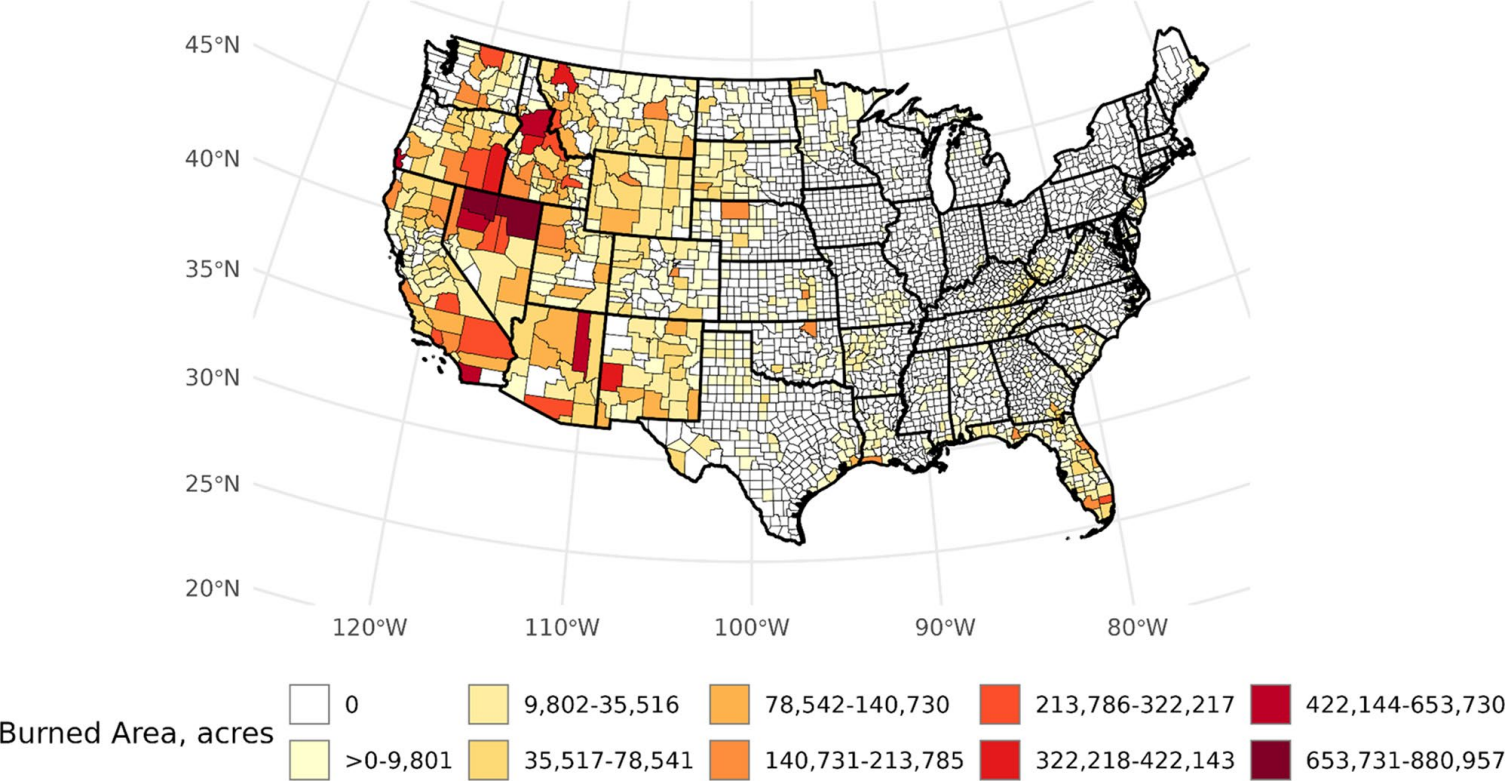
Distribution of area burned across conterminous US counties, 1997–2003

Between 2016 and 2020, a total of 774,466 adult lung cancer deaths occurred, with female- and male-specific age-adjusted LCM rates of 51.1 and 64.0 cases per 100,000 persons, respectively (Table 2). Rates were highest among females in the Eastern USFS region. We observed higher rates for women in specific counties in Tennessee, Kentucky, Virginia, Nebraska, and Georgia (Figure 2). The highest LCM rates among men were observed in the Southern USFS region, with wide variability across multiple states.

**Table 2:** Lung cancer mortality (LCM) rates [1] across the conterminous United States (US), 2016–2020, overall and by US forest service regions [2]

Lung and bronchus mortality	Overall	Eastern	Intermountain	Northern	Pacific Northwest	Pacific Southwest	Rocky Mountain	Southern	Southwestern
**Deaths**,** females**	357,912	151,762	8,325	2,975	12,434	30,617	12,228	125,994	13,577
**Mortality rate**^**1**^, **females**, **(95% CI)** ^**3**^	51.1 (50.8–51.2)	75.3 (74.6–75.4)	49.8 (48.9–51.1)	68.2 (65.6–70.4)	66.1 (64.8–67.2)	56.0 (55.4–56.6)	67.7 (66.8–69.2)	74.5 (73.6–75.4)	46.6 (46.2–47.8)
**Residuals**,** ever smoker LCM rate**,** females**,** (SD)**^**4**^	0.015 (0.023)	0.673 (0.037)	-2.09 (0.477)	-0.161 (0.041)	0.837 (0.307)	1.14 (0.569)	-0.399 (0.204)	0.457 (0.059)	-1.32 (0.371)
**Residuals**,** current smoker LCM rate**,** females**,** (SD)**^**4**^	0.080 (0.024)	0.319 (0.040)	-1.75 (0.344)	-0.690 (0.169)	-0.905 (0.276)	-1.06 (0.528)	-0.475 (0.266)	1.600 (0.075)	-0.672 (0.214)
**Deaths**,** males**	416,554	168,569	9,024	3,282	13,012	33,448	13,692	160,302	15,225
**Mortality rate**^**1**^, **males**, **(95% CI)** ^**3**^	64.0 (63.8–64.2)	95.1 (94.5–95.5)	54.5 (53.9–56.1)	76.5 (74.4–79.6)	72.6 (71.7–74.3)	60.3 (59.4–60.6)	82.9 (81.6–84.4)	106.6 (106.5-107.5)	58.0 (57.1–58.9)
**Residuals**,** ever smoker LCM rate**,** males**,** (SD)**^**4**^	0.205 (0.027)	1.37 (0.063)	-1.86 (0.417)	0.165 (0.058)	0.504 (0.162)	-1.630 (0.816)	-0.19 (0.244)	0.944 (0.100)	-1.500 (0.346)
**Residuals**,** current smoker LCM rate**,** males**,** (SD)**^**3**^	-0.284 (0.022)	-0.418 (0.051)	-1.19 (0.305)	-1.10 (0.241)	-1.480 (0.430)	-2.080 (1.038)	-0.675 (0.191)	1.050 (0.058)	-0.089 (0.094)

^1^ Per 100,000 population

^2^ Area-weighted estimates

^3^ Aggregated sex-specific rates are age-adjusted based on the 2000 US standard population

^4^ Deviance residuals derived from Poisson regression fit between county-level cigarette smoking and county-level sex-specific, adjusted lung cancer mortality rates

**Fig. 3:**
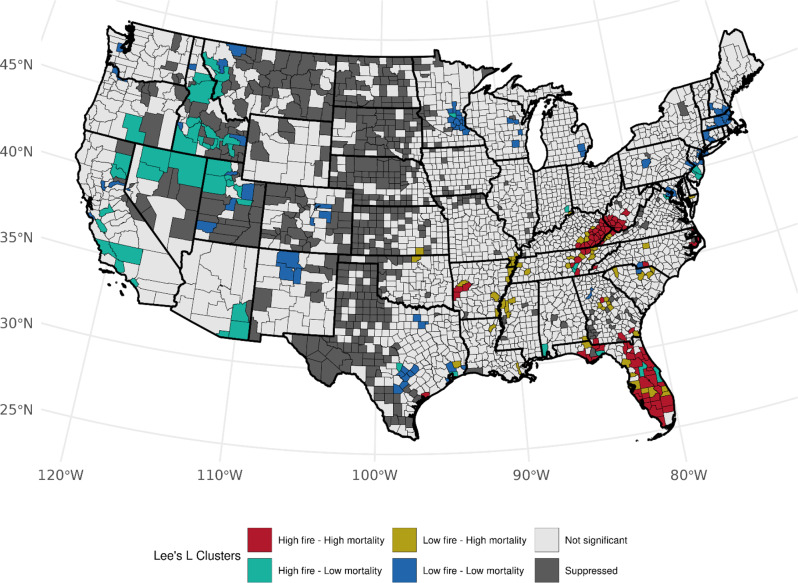
Bivariate associations between county-level wildland fire density (# events per 1,000 km squared, 1997–2003) and age-adjusted LCM rates (2016–2020) among females, accounting for the county-level prevalence of ever smoking (1997–2003). Not significant denoted as *p* > 0.05

**Fig. 4:**
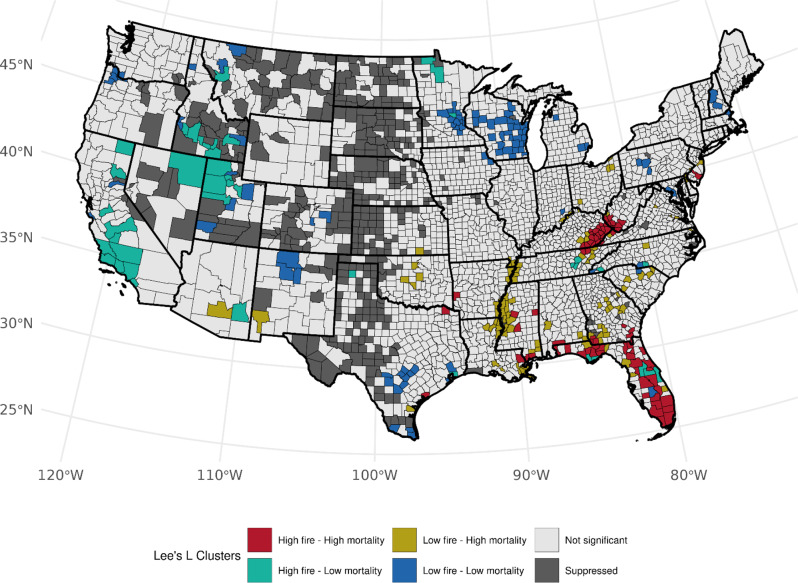
Bivariate associations between county-level wildland fire density (# events per 1,000 km squared, 1997–2003) and age-adjusted LCM rates (2016–2020) among males, accounting for the county-level prevalence of ever smoking (1997–2003). Not significant denoted as *p* > 0.05

Accounting for the prevalence of ever-smoking, we observed over 82 counties (Table S-1) with high area-based WF density and high LCM rates (high-high) among females, mainly across Florida (*n* = 32, *p*-values: < 0.0005–0.030), Kentucky (*n* = 20, *p*-values: < 0.0005–0.014), and West Virginia (*n* = 10, *p*-values: 0.0012–0.044), and several clusters also observed in Tennessee, North Carolina, Georgia, and Arkansas (Fig. 3). An association with the opposite pattern of low WF density and high LCM was found across 54 counties with Florida (*n* = 9, *p*-values: 0.006–0.049), Tennessee (*n* = 8, *p*-values: 0.001–0.049, and Georgia (*n* = 6, *p*-values: 0.013–0.033) having the most clusters. We also observed notable low-high clustering along the Mississippi River. Among males, we observed a consistent number of high-high counties in Florida (*n* = 28, *p*-values: < 0.0005–0.049), Kentucky (*n* = 18, *p*-values: < 0.0005–0.028), West Virginia (*n* = 7, *p*-values < 0.0005–0.009), and Georgia (*n* = 6, *p*-values: 0.0048–0.049) with observed fewer counties in Mississippi, Texas, and New Jersey. (Fig. 4). We found changes in cluster types when accounting for the prevalence of current-smoking for both sexes (Table S-1); however, patterns specifically within Florida and Kentucky were consistent for both sexes within West Virginia, and among males when accounting for ever-smoker prevalence (Figures S4 and S5). Patterns for high-low clustering within the western states were also similar except for a few high-high counties within California and Arizona.

**Fig. 5:**
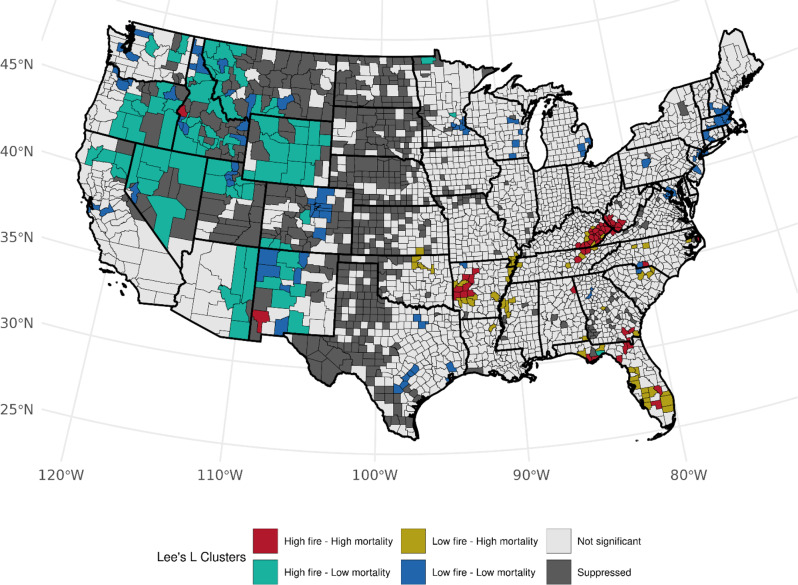
Bivariate associations between population-normalized county-level wildland fire density (# events per 100,000 persons, 1997–2003) and age-adjusted LCM rates (2016–2020) among females, accounting for the county-level prevalence of ever smoking (1997–2003). Not significant denoted as *p* > 0.05

**Fig. 6:**
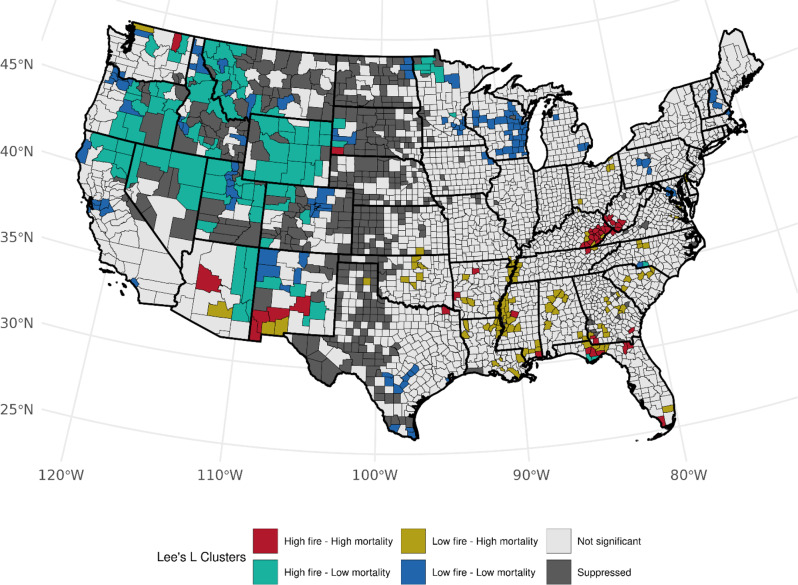
Bivariate associations between population-normalized county-level wildland fire density (# events per 100,000 persons, 1997–2003) and age-adjusted LCM rates (2016–2020) among males, accounting for the county-level prevalence of ever smoking (1997–2003). Not significant denoted as *p* > 0.05

When considering population-based WF density, 50 counties had high WF density - high LCM rate results among females (Table S-1). Kentucky (*n* = 17, *p*-values: 0.002–0.034), Arkansas (*n* = 8, *p*-values: < 0.005–0.036), West Virginia (*n* = 7, *p*-values: 0.015–0.033), and Florida (*n* = 7, *p*-values: 0.003–0.048) made up the majority of this characteristic (Fig. 5). Fifty-four counties exhibited low WF density - high LCM clustering in Florida (*n* = 14, *p*-values: 0.011–0.048) and Arkansas (*n* = 9, *p*-values: 0.014–0.046) and also along the Mississippi River (*n* = 11, *p*-values: 0.030–0.048). Among males and accounting for ever-cigarette smokers, similar high-high clustering in Kentucky, West Virginia, and Florida was noted, with fewer counties in Arkansas and more in New Mexico (*n* = 4, *p*-values: 0.001–0.025) (Fig. 6). High-high cluster patterns across Florida, Kentucky, and West Virginia were somewhat reduced using the LCM residuals adjusted by current-smoking prevalence. Despite the prominent presence of high-low clusters, we observed increased high-high clustering across the Western region, such as California and Oregon for females and males (Figures S-6 and S-7) and Minnesota for males (Figure S-7).

**Fig. 7:**
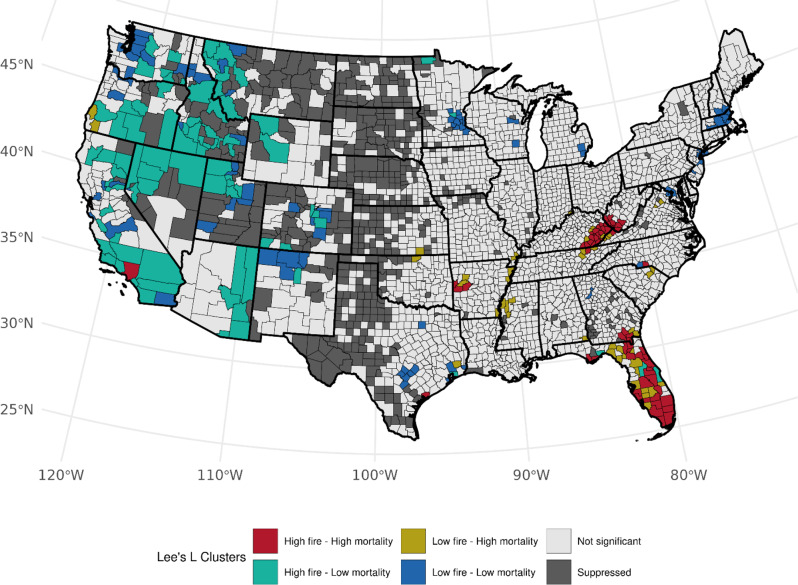
Bivariate associations between county-level burned area in a county-to-area county ratio (1997–2003) and age-adjusted LCM rates (2016–2020) among females, accounting for the county-level prevalence of ever smoking (1997–2003). Not significant denoted as *p* > 0.05

**Fig. 8:**
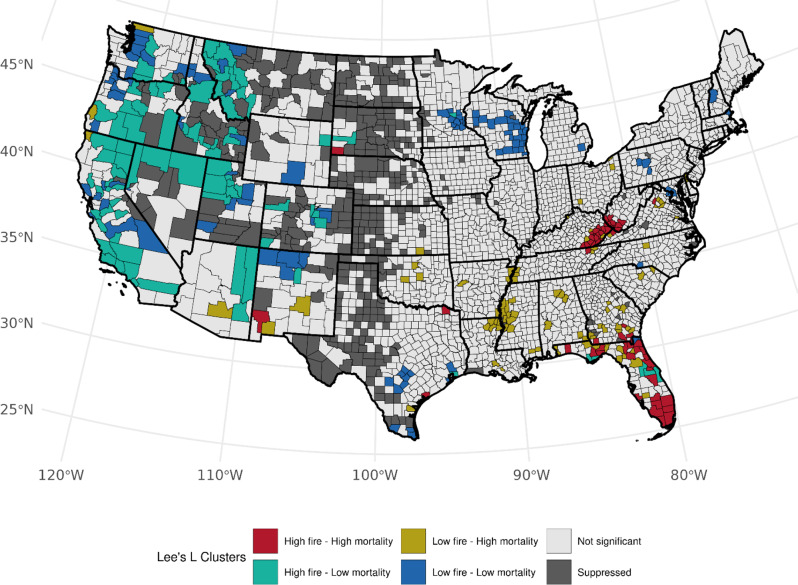
Bivariate associations between county-level burned area in a county-to-area county ratio (1997–2003) and age-adjusted LCM rates (2016–2020) among males, accounting for the county-level prevalence of ever smoking (1997–2003). Not significant denoted as *p* > 0.05

For the burn area ratio exposure metric, when accounting for ever-smoker prevalence, we observed the same high-high clustering among females in Florida (*n* = 25, *p*-values: < 0.0005–0.041), Kentucky (*n* = 16, *p*-values: < 0.0005–0.017) and West Virginia (*n* = 7, *p*-values: 0.009–0.032), and also clusters in Arkansas, Georgia, and Tennessee, and counties in California and Texas (Fig. 7). High-low associations were found in California (*n* = 17, *p*-values: < 0.0005–0.047), Idaho (*n* = 13, *p*-values = 0.0005–0.0431), Montana (*n* = 7, *p*-values: < 0.0005–0.031), and other western states including Arizona, Colorado, Nevada, Utah, and Washington. We observed general concordance with these clusters among males. Similar to females, high-high clusters among males were found in Florida (*n* = 22, *p*-values: < 0.0005–0.044), Kentucky (*n* = 15, *p*-values: < 0.0005–0.048), and West Virginia (*n* = 7, *p*-values: 0.004–0.035) with smaller clusters also found in Georgia. (Fig. 8). Most high-low clusters were found in California (*n* = 22, *p*-values: < 0.0005–0.046), Montana (*n* = 10, *p*-values: 0.003–0.025), Idaho (*n* = 9, *p*-values: 0.004–0.030), Oregon (*n* = 9, *p*-values: 0.001–0.044), and Utah (*n* = 7, *p*-values: < 0.0005–0.015) (Fig. 8). We observed similar, however, modestly reduced, high-high cluster patterns for the burn area ratio exposure metric when accounting for the prevalence of current cigarette smoking. At the state level, a reduction of high-high clusters was observed across Kentucky and West Virginia with the introduction of a few high-low clusters. Also, more high-high counties across the western region were detected when accounting for current smoker prevalence (Figures S-8 and S-9) within the California-Nevada-Oregon border region.

**Fig. 9:**
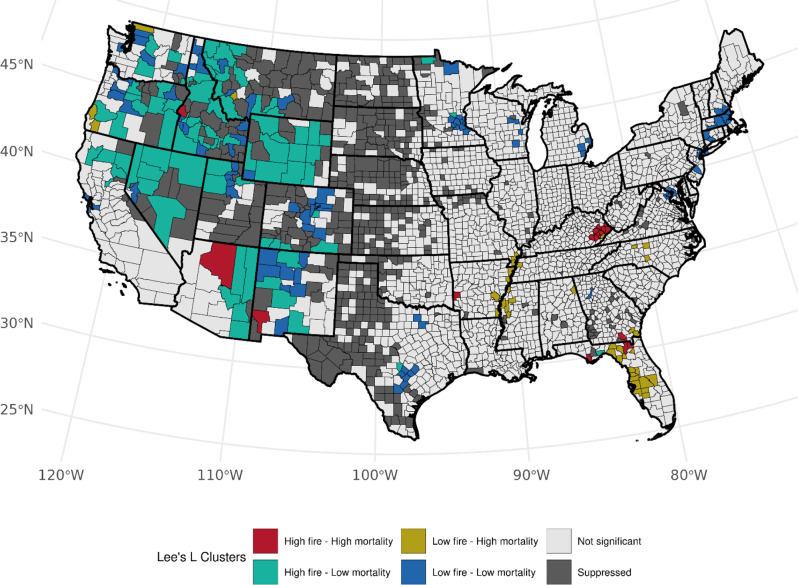
Bivariate associations between county-level burned area per 100,000 persons (1997–2003) based on county population and age-adjusted LCM rates (2016–2020) among females, accounting for the county-level prevalence of ever smoking (1997–2003). Not significant denoted as *p* > 0.05

**Fig. 10:**
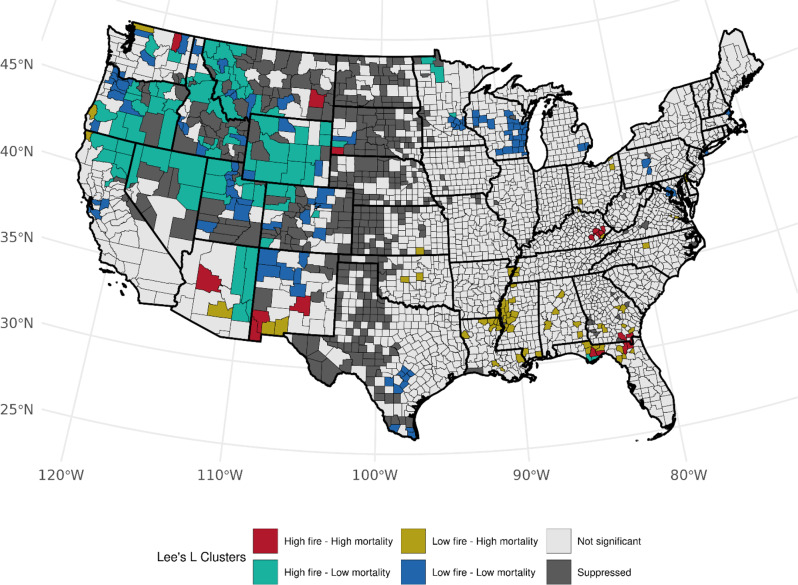
Bivariate between county-level burned area per 100,000 persons (1997–2003) based on county population and age-adjusted LCM rates (2016–2020) among males, accounting for the county-level prevalence of ever smoking (1997–2003). Not significant denoted as *p* > 0.05

Lastly, seventeen high-high clusters were detected based on analyses using population-based burned area ratio while accounting for ever smoking among females (Table S-1), with most clusters in Kentucky (*n* = 9, *p*-values: 0.016–0.049) and Florida (*n* = 3, *p*-values: 0.014–0.046), and other significant counties dispersed across the Western region (Fig. 9). Nearly 40 counties showed low-high clustering, primarily in Florida (*n* = 16, *p*-values: 0.012–0.046) and along the Mississippi River (*n* = 12, *p*-values: 0.033–0.049). Among males and accounting for ever-smokers, high-high clustering in Kentucky (*n* = 5, *p*-values: 0.019–0.036), Florida (*n* = 4, *p*-values: 0.013–0.034), New Mexico (*n* = 3, *p*-values: < 0.0005–0.039), and Georgia (*n* = 2, *p*-values: 0.032–0.036) is shown (Fig. 10). After accounting for current-smokers, we found differences in high-high cluster distributions between ever-smoker and current-smoker-adjusted (Figures S10 and S-11) rates. We observed cluster reductions across Florida, Kentucky, and West Virginia, with more clusters appearing in western states, notably California and Oregon. A decrease in low-high clusters along the Mississippi River was observed for both sexes.

After FDR correction, we observed reduced clustering across CONUS for each exposure metric (Figures S-12 - S-15). No high-high clusters were observed in the Western USFS region. High-high clusters between WF density and adjusted LCM rates among females and males were mostly concentrated in Florida, and among females in Kentucky after adjusting for ever-smoker prevalence (*n* = 5, *p*-values: < 0.0005). Patterns within Florida were similar for current-smoker-adjusted analyses using the county-area normalized burned-area ratio. Almost all analyses of population-based exposure metrics yielded few high-low clusters within western states irrespective of biological sex and smoking adjustment.

## Discussion

In this ecologic analysis, we observed varying patterns of bivariate associations between large WFs and LCM rates across US counties while accounting for cigarette smoking prevalence. Specifically, we found consistent positive associations for males and females in counties clustered within the mid-Appalachian region and Florida, a finding previously unreported in the literature. Similar patterning was observed between males and females with high-low cluster types across several western counties. Our finding of positive bivariate relationships between WF metrics and LCM rates for both sexes in the Appalachian region was interesting given the dominance of the western region in most wildfire investigations to date.

Wildland fires within the Appalachian region, particularly in eastern Kentucky and West Virginia, include heavily forested regions (e.g., Cumberland Mountains and Daniel Boone National Forest) that are susceptible to larger (> 500 acres) wildfire occurrences during drier conditions in the autumn seasons. Higher yield of accumulated oak-dominated leaf litter biomass in higher elevations and median slopes are conducive to increased fire occurrences and intensities [40, 41]. Regionally, human-caused ignitions were the primary causes of wildfires within the Cumberland Mountains between 1985 and 2005 [40]. 

In Florida, MTBS data between 1997 and 2003 show that causes of wildland fires include wildfires (*n* = 156), prescribed burns (*n* = 42), wildland fire use (*n* = 5), and unknown (*n* = 313; cause of fire not reported, undetermined, or fire origin is destroyed) [6]. These fire events occurred year-round and mostly during the dry season from October to May, with a notable spike in wildfires (*n* = 30) in June. A study using point pattern clustering analysis found that in addition to crop burning, arson, lightning strikes, and railroads are likely causes of fire ignition in Florida [42]. Prescribed crop burns from sugarcane production constitute a significant source of airborne particulate matter and are positively associated with premature deaths within Southern Florida [43]. Emissions from sugarcane burning include numerous pollutants similar to wildfire ash, such as carcinogenic PAHs, but with higher silica content [44]. In contrast to some of the drier regions in the western US, the increased soil moisture and humid conditions in Florida allow for more rapid vegetation recovery and regrowth following a fire event and, consequently, increasing fuel for future wildfires and prescribed burns over shorter periods, particularly within tropical and subtropical savanna landscapes [45, 46]. 

Prior work has demonstrated higher smoking rates and high lung cancer mortality in both sexes within this same Appalachian region, spanning from eastern Tennessee to West Virginia [47]. Historically, higher LCM rates have been documented within Appalachian states such as Kentucky, with cigarette smoking and coal-mining-related exposures as potential explanatory factors, especially among men [48, 49]. One study applied a spatial scan statistic in Kentucky and identified clusters of high lung cancer incidence rates in regions known for coal production after adjusting for smoking status, age, and sex [50]. Notably, one of the findings involved a cluster of southeastern counties with a 21% higher rate of lung cancer incidence compared to the entire state. Our analyses did not explicitly account for other non-WF environmental exposures, such as emissions from coal mining, and we acknowledge they may have contributed to our observed associations.

We observed low-high clustering along the Mississippi River Delta across each exposure metric and sex type when accounting for ever smoking prevalence. In a previous study focusing on geospatial clustering between cigarette smoking prevalence and lung cancer mortality rates, researchers reported a similar pattern [47]. They speculated that this patterning might be due to environmental risk factors, such as industrial or agricultural activity in the Delta.

Our analysis revealed positive associations between WF and lung cancer mortality across several Western US counties that include prominent wildfire-impacted states such as New Mexico, California, Oregon, and Idaho. In a more recent study in California, chronic occupational smoke exposure was linked to increased risk of lung cancer and cardiovascular disease mortality among wildland firefighters [51]. After accounting for multiple comparisons, we noted a substantial decrease in clusters in our analysis, particularly within the Western region. This finding is interesting given the relatively greater burden of WF exposures in this part of the country. However, some positive clustering remained around Florida and the Appalachian region, specifically within Kentucky. We speculate that a few factors could potentially explain these observations. For example, adjusting for multiple comparisons likely reduced our power to detect statistically significant associations. As such, investigating these relationships using individual-level data with analytic study designs may be necessary to fully interrogate the association between WF and LCM within high-high counties. We also acknowledge that these results could be due to chance. However, we suggest that the consistency in geographic patterns we observed for both sexes across multiple exposure metrics and different cigarette smoking adjustments argue against this as fully explaining our findings.

We applied an indirect approach to incorporating cigarette smoking prevalence, the predominant lung cancer risk factor, in our analysis by focusing on the deviation between regressed cigarette smoking and LCM rates across counties. As an exploratory spatial analysis focused primarily on bivariate patterning, thi provided an efficient approach to adjusting for smoking between WF and LCM rates to account for county-level smoking prevalence. We did observe some differences in patterns of association when accounting for current versus ever smoking prevalence, particularly that clusters were sometimes not detected in current smoking-adjusted models. This may be an artifact of the reduced power of these analyses, given that current smoking prevalence may be more strongly associated with LCM rates than ever smoking. However, future studies with individual data on smoking use and wildfire exposure are needed to extend these findings.

We are unaware of other studies exploring geospatial variability in the association between long-term WF exposure and LCM within the US. However, some studies have used residential proximity to wildland fires as a long-term exposure proxy and also modeled wildfire-derived air pollutant measures in their exposure assessments [52]. We did not consider smoke plume exposures or possible long-range smoke plumes from individual wildland fire sites. It is possible that downstream counties with no reported fire occurrences through MTBS could have been impacted by the transport of smoke plumes. However, our use of location-specific wildfire occurrences within counties provided reliable assurance that wildland fire-derived pollutants were likely distributed across region-specific populations, as noted in other studies [30]. A recent review identified 17 published epidemiologic studies of the long-term health effects of wildfires, with only two involving cancer risk. Most investigations outside occupational settings have focused on short-term WF exposures resulting in cause-specific emergency department visits [15, 16], hospital admissions [17], and mortality [18] from multiple causes [53]. One study characterized survivability among individuals receiving treatment for lung cancer tissue resection across the US and found overall increased mortality following wildfire exposure for up to a month after hospital discharge [32]. Taken together, while the pre-existing literature shows that acute health effects are increasingly associated with wildland fire exposures, chronic health conditions such as cancer remain understudied.

Our analyses incorporated multiple WF exposure metrics normalized by county-level area and population size. In prior studies, area-based fire density has been used to represent fire occurrences [40], and we used an area-based density measure to account for variability in county size. We also incorporated population density-based metrics to similarly account for differences in underlying county population sizes. Overall, both area- and population-density WF metrics yielded similar geographic patterning in relation to lung cancer mortality rates, irrespective of sex and cigarette smoking status. In addition to employing analytic study designs, future studies may benefit from improving the specificity of exposure metrics, such as using WF-derived air emissions (e.g., PM_2.5_) from long-term exposure assessments and across smoke plume-impacted regions, to investigate these associations.

Our study offered major strengths, including using high-quality open-source WF data by the largest and most comprehensive post-fire mapping and assessment programs in the US. We accounted for a minimum of 13 years of latency between WF exposures and lung LCM. Importantly, our analyses adjusted for cigarette smoking prevalence, suggesting that our findings for WF may be independent of this important lung cancer risk factor, which is estimated to cause over 80% of lung cancer deaths in the US population. Our study design prevented us from directly controlling for confounding, so we applied an indirect approach to address the influence of cigarette smoking prevalence on our observed associations by focusing on the deviation between regressed cigarette smoking and LCM rates across counties. As an ecologic study focused on geospatial patterning, this provided an efficient approach to adjusting for smoking. We also accounted for multiple comparisons to ensure control over Type I errors.

We note some limitations to this study. Our ecologic design relied on data aggregated to the county level, which precluded adjustment for potential individual-level confounders, including occupation, socioeconomic status, and prevailing wind direction; as such, our interpretations are intended to inform future, more detailed investigations designed for hypothesis testing. We also focused on an exposure period from 1997 to 2003, driven largely by the need to coincide with the earliest available cigarette smoking prevalence data. This limited our ability to consider earlier and broader time scales. Our analysis did not consider wildland fire events with burned areas of less than 500 and 1,000 acres in the eastern and western portions of the US, respectively, which may have led to the exclusion of some smaller fires. However, given that 95% of the annualized burned area is captured through MTBS, we expect the impact of any resulting exposure misclassification to be minimal. Additionally, smoking frequency and duration also influence LCM and may vary spatially, but were not incorporated in our analysis. A longitudinal study design might be better suited for elucidating the etiologic role of wildland fires in lung cancer mortality and characterizing temporal variability in wildland fire exposures.

## Conclusion

Our analysis identified counties both within and outside the western US where wildland fire exposures may contribute to LCM. Studies with individual-level exposure-response assessments are needed to evaluate this relationship further, and future evaluation of additional cancer sites would be informative, particularly those also linked to PM_2.5_ exposure. This work adds new information to understanding the potential role of larger-scaled WFs across the conterminous US as an environmental determinant for lung cancer.

## Electronic supplementary material

Below is the link to the electronic supplementary material.

Supplementary Material 1

## Data Availability

No datasets were generated or analysed during the current study.
