# Supplementary material for: Geographic patterns in wildland fire exposures and county-level lung cancer mortality in the United States
Source: Int J Health Geogr. 2025 Apr 11;24:8. doi: 10.1186/s12942-025-00394-x (PMC11992742; doi:10.1186/s12942-025-00394-x)
Supplement: Supplementary file 1 — Supplementary Material 1 [file 12942_2025_394_MOESM1_ESM.docx]

**Supplemental Tables and Figures**

**Table S-1.** The number of counties in clusters by exposure metric type, and sex, and cigarette smoking type. Counties are enumerated for high-high, high-low, low-high, low-low, not statistically significant (NS), and suppressed categories.

| **Metric** | **Type** | **Sex** | **Smoking Status** | **High-High** | **High-Low** | **Low-High** | **Low-Low** | **NS** | **Suppressed** |
| --- | --- | --- | --- | --- | --- | --- | --- | --- | --- |
| **Fire Density** | **Area** | **Female** | **Ever** | 82 | 49 | 54 | 89 | 2,303 | 531 |
|  |  |  | **Current** | 51 | 41 | 42 | 77 | 2,366 | 531 |
|  |  | **Male** | **Ever** | 75 | 42 | 98 | 103 | 2,382 | 408 |
|  |  |  | **Current** | 57 | 53 | 34 | 94 | 2,462 | 408 |
|  | **Population** | **Female** | **Ever** | 50 | 86 | 54 | 103 | 2,284 | 531 |
|  |  |  | **Current** | 19 | 74 | 56 | 81 | 2,347 | 531 |
|  |  | **Male** | **Ever** | 44 | 91 | 102 | 116 | 2,347 | 408 |
|  |  |  | **Current** | 19 | 74 | 52 | 104 | 2,451 | 408 |
| **Burned Area** | **Area** | **Female** | **Ever** | 59 | 82 | 46 | 96 | 2,294 | 531 |
|  |  |  | **Current** | 55 | 63 | 46 | 71 | 2,342 | 531 |
|  |  | **Male** | **Ever** | 55 | 83 | 85 | 106 | 2,371 | 408 |
|  |  |  | **Current** | 40 | 75 | 32 | 102 | 2,451 | 408 |
|  | **Population** | **Female** | **Ever** | 17 | 82 | 39 | 111 | 2,328 | 531 |
|  |  |  | **Current** | 18 | 62 | 47 | 79 | 2,371 | 531 |
|  |  | **Male** | **Ever** | 18 | 79 | 66 | 110 | 2,427 | 408 |
|  |  |  | **Current** | 13 | 76 | 28 | 99 | 2,484 | 408 |

**
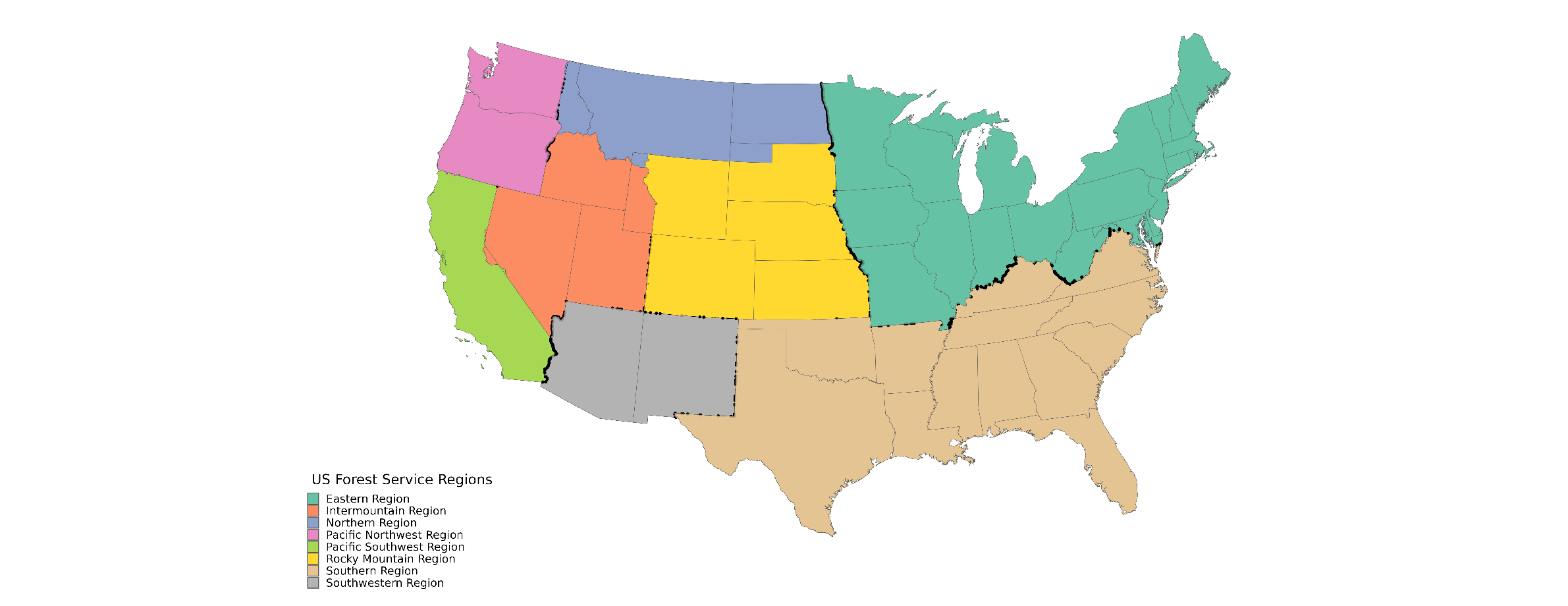
**

**Figure S-1.** The United States (US) Forest National Forest System regions across the conterminous US.


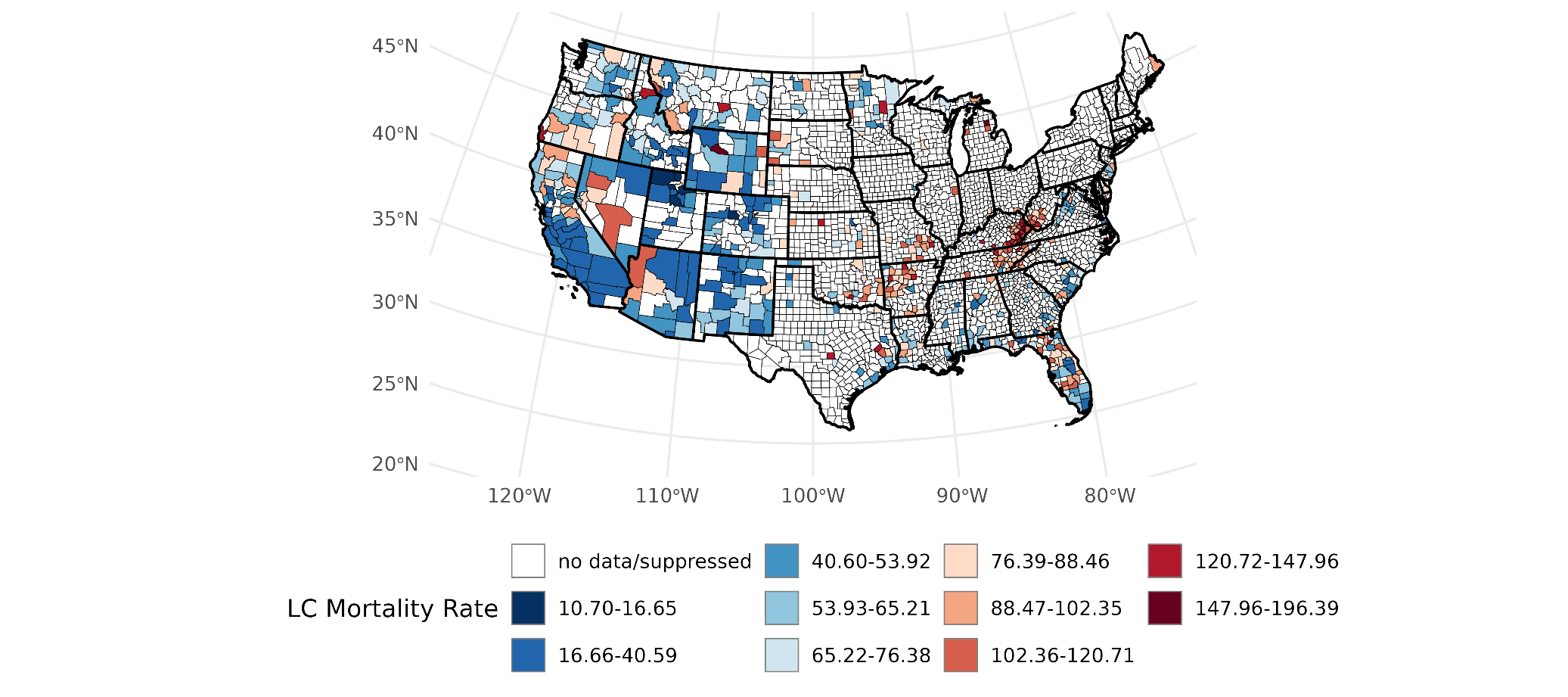
**Figure S-2** Choropleth map of conterminous US counties characterizing spatial distribution age-adjusted, female lung cancer mortality rates from 2016-2020, lung cancer (LC) mortality cases per 100,000 females.


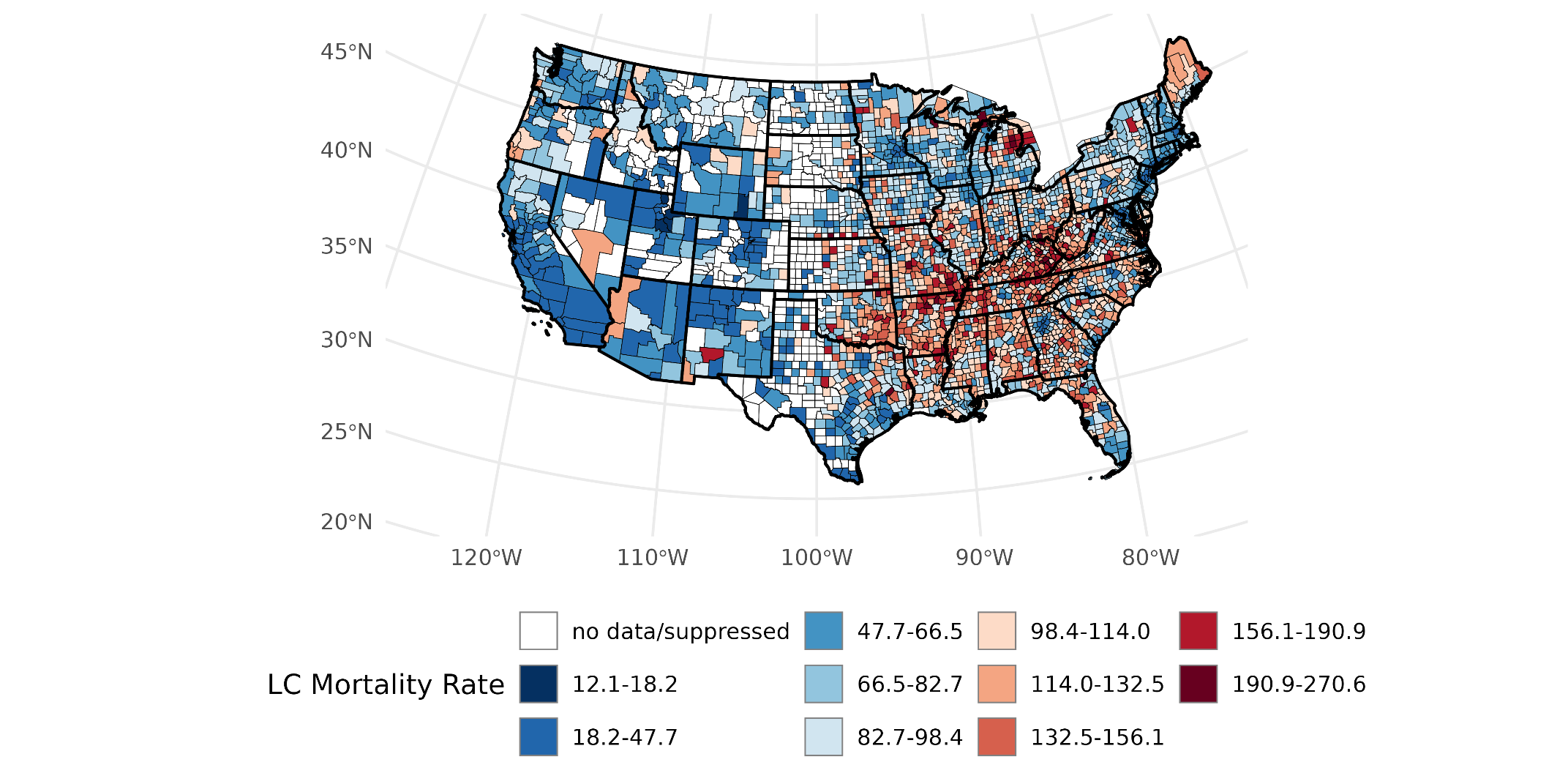


**Figure S-3** Choropleth map of conterminous US counties characterizing spatial distribution age-adjusted, male lung cancer mortality rates from 2016-2020, lung cancer (LC) mortality cases per 100,000 males.

| 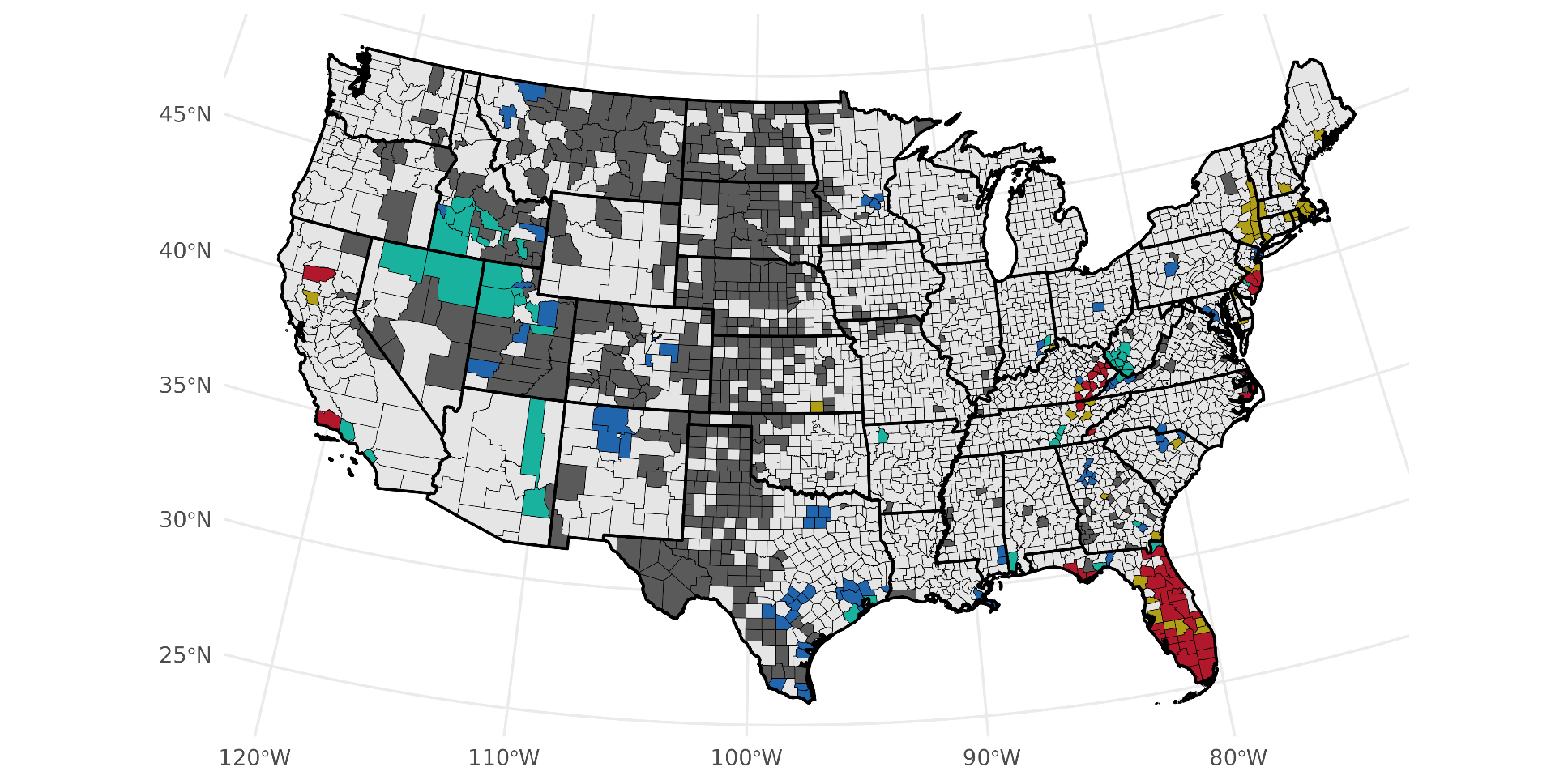 |
| --- |
| 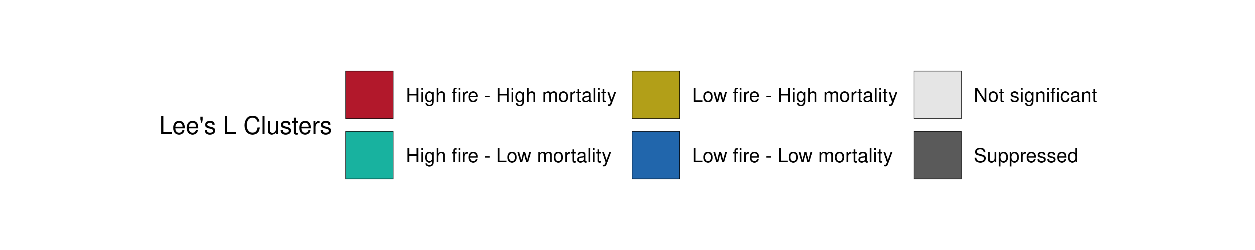 |

**Figure S-4** Bivariate associations between county-level wildland fire density (# events per 1,000 km^2^, 1997-2003) and age-adjusted LCM rates (2016-2020) among females, accounting for county-level prevalence of current smoking. Not significant denoted as *p* > 0.05.

| 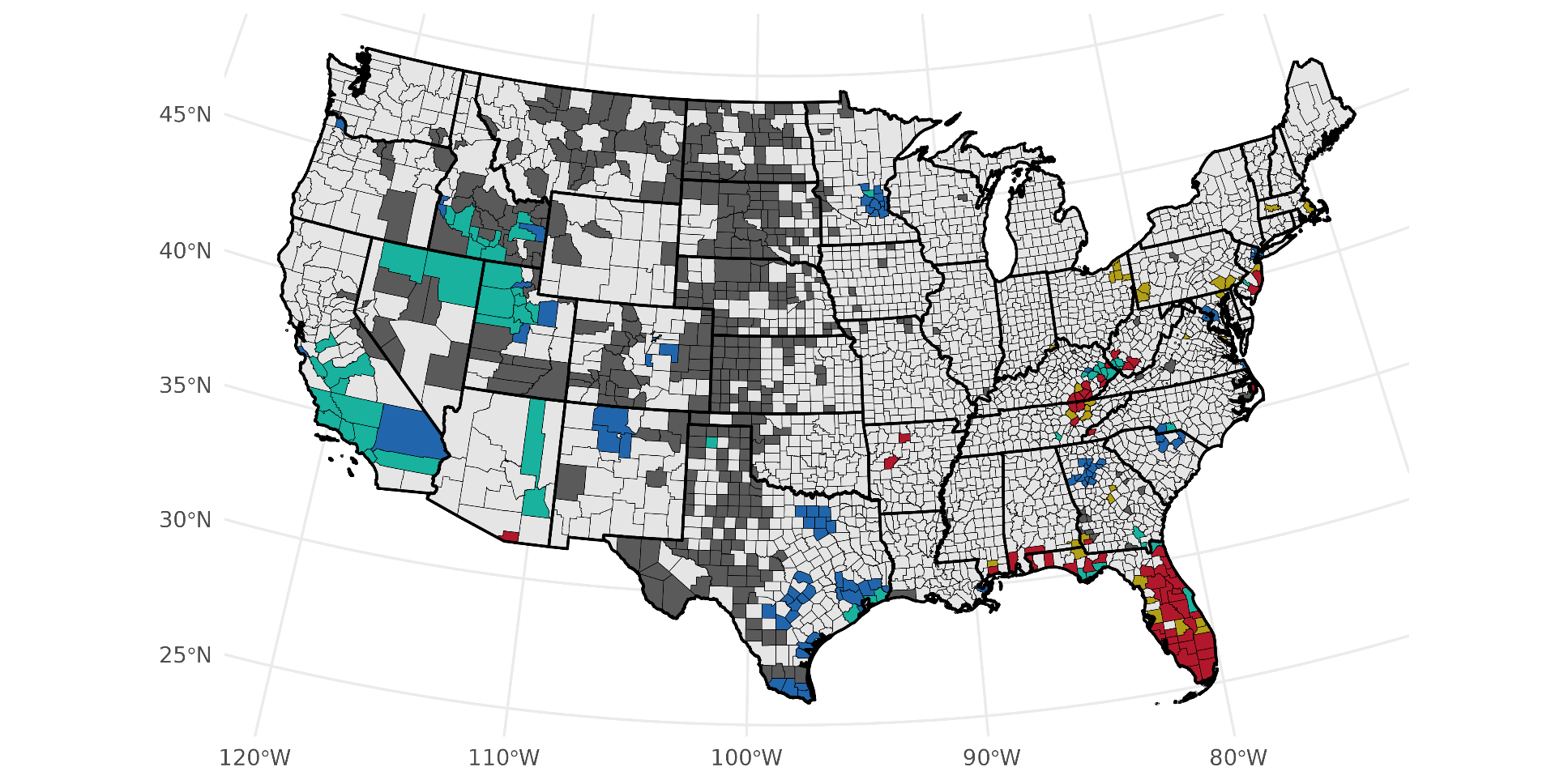 |
| --- |
| 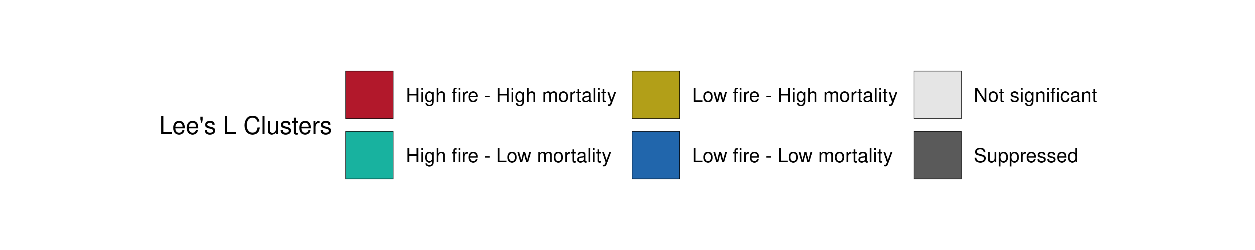 |

**Figure S-5** Bivariate associations between county-level wildland fire density (# events per 1,000 km^2^, 1997-2003) and age-adjusted LCM rates (2016-2020) among males, accounting for county-level prevalence of current smoking. Not significant denoted as *p* > 0.05.

| 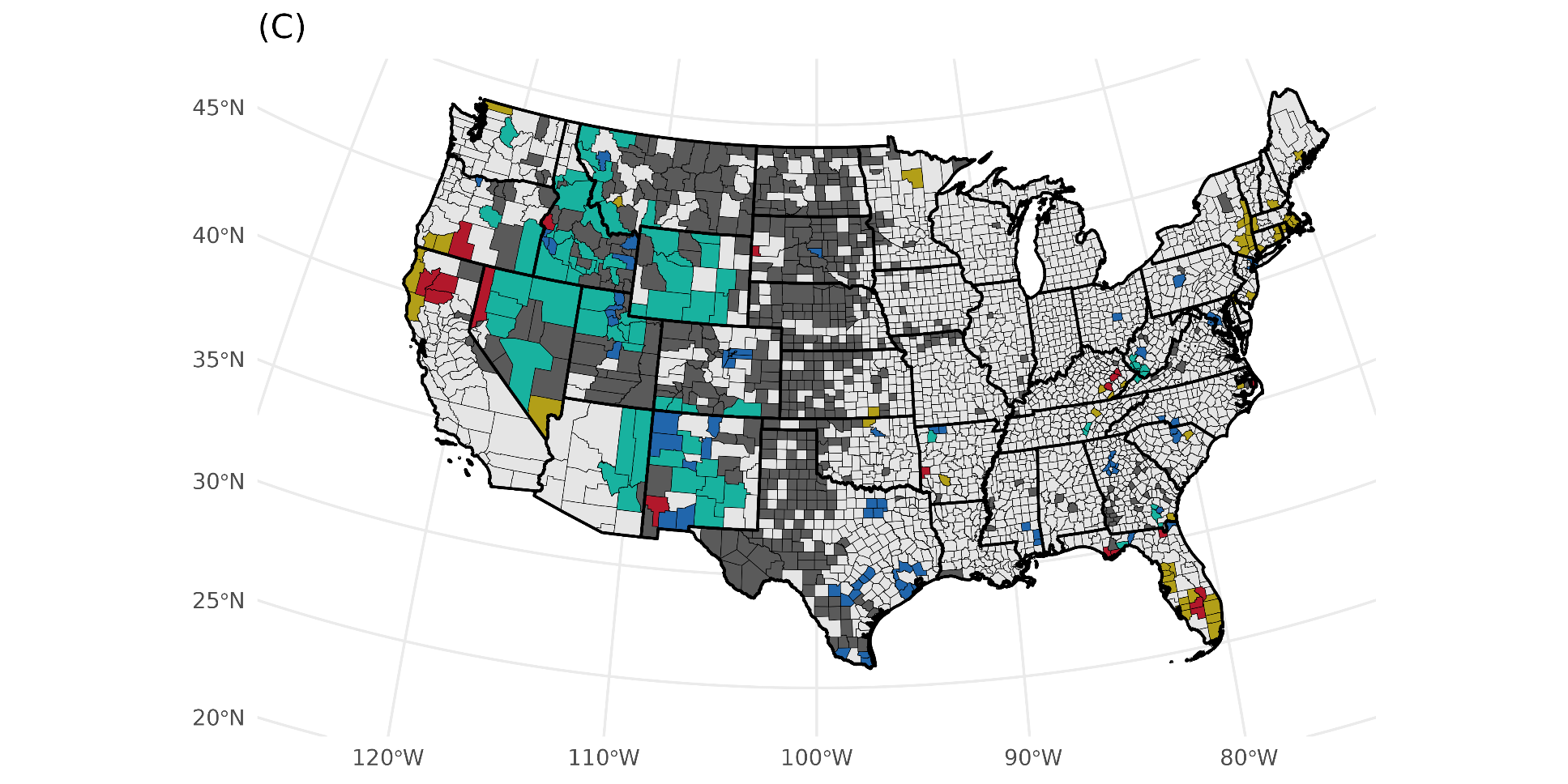 |
| --- |
| 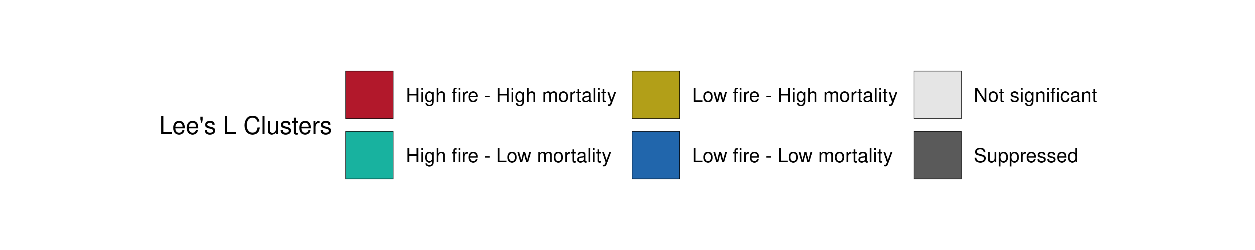 |

**Figure S-6** Bivariate associations between population-normalized county-level wildland fire density (# events per 100,000 persons, 1997-2003) and age-adjusted LCM rates (2016-2020) among females, accounting for county-level prevalence of current smoking. Not significant denoted as *p* > 0.05.

| 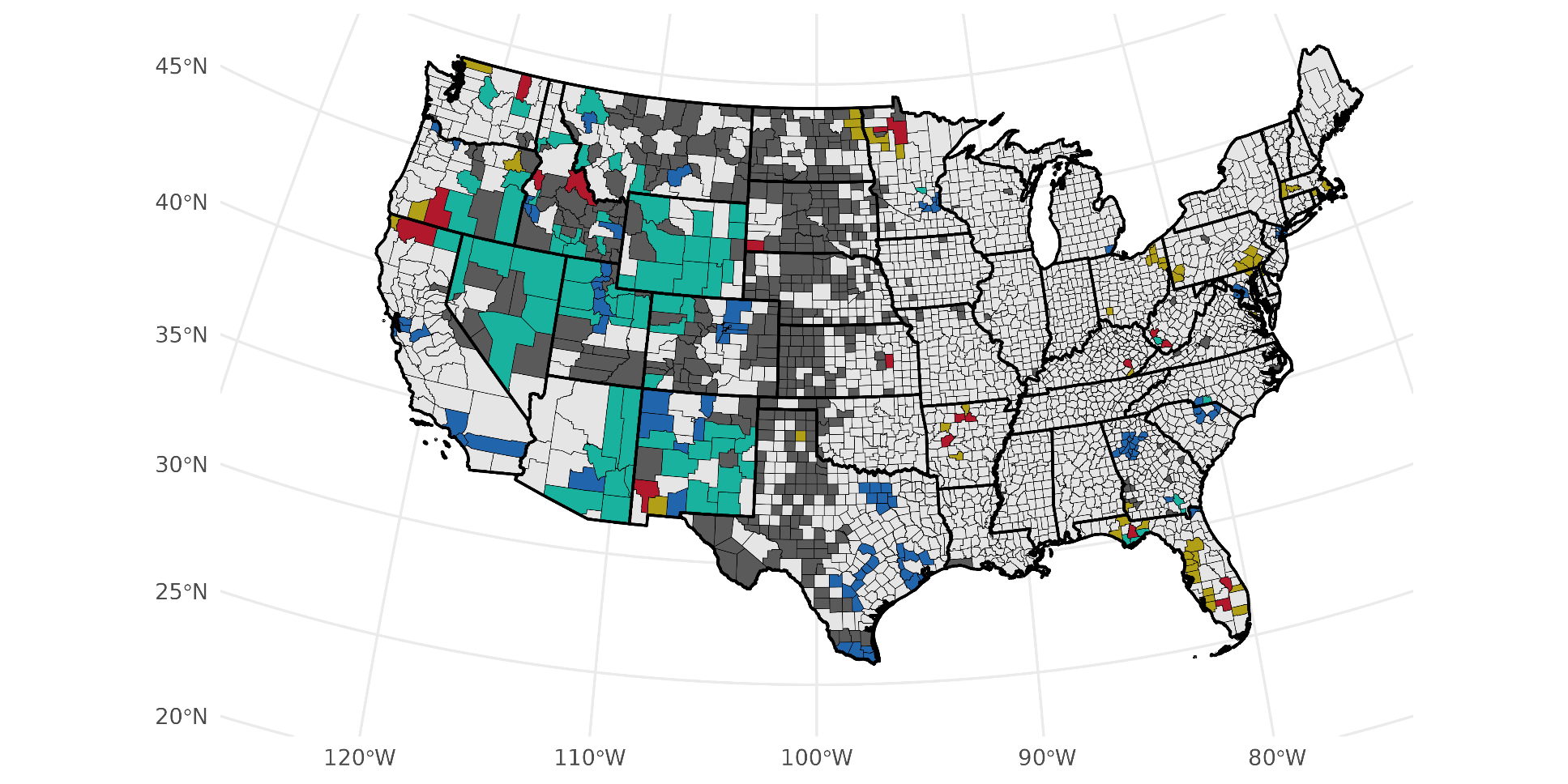 |
| --- |
| 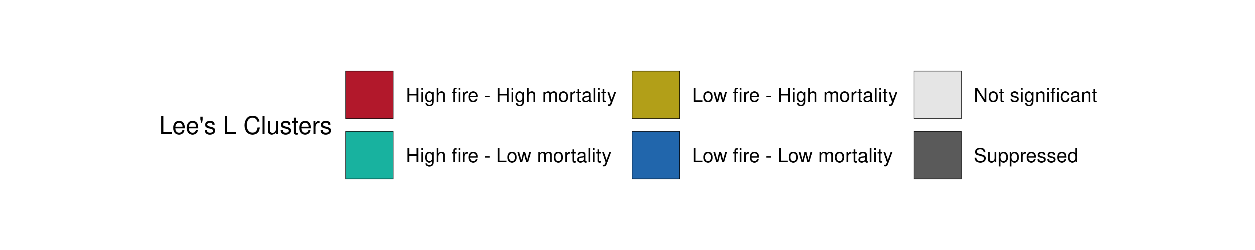 |

**Figure S-7** Bivariate associations between population-normalized county-level wildland fire density (# events per 100,000 persons, 1997-2003) and age-adjusted LCM rates (2016-2020) among males, accounting for county-level prevalence of ever smoking. Not significant denoted as *p* > 0.05.

| 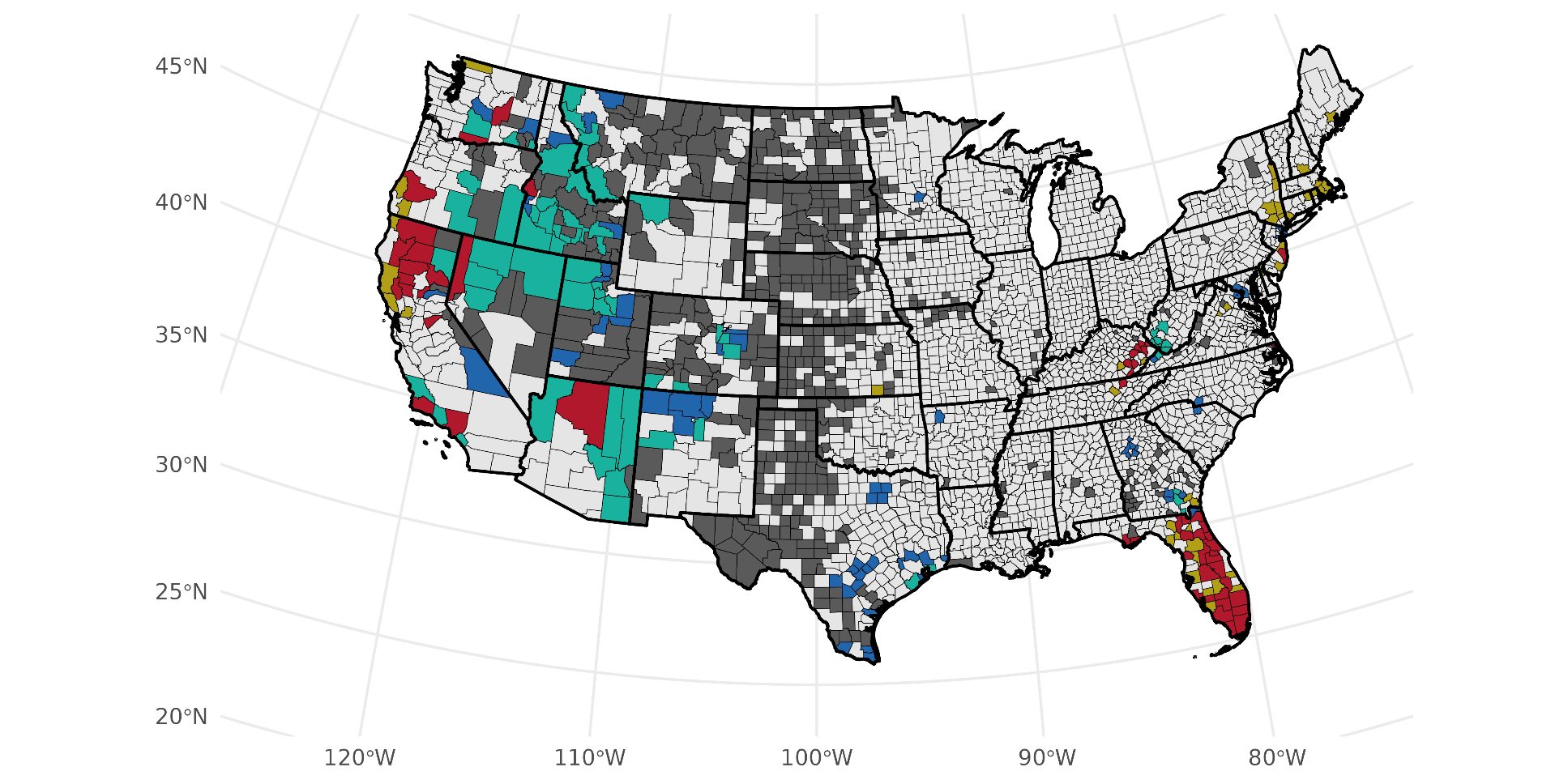 |
| --- |
| 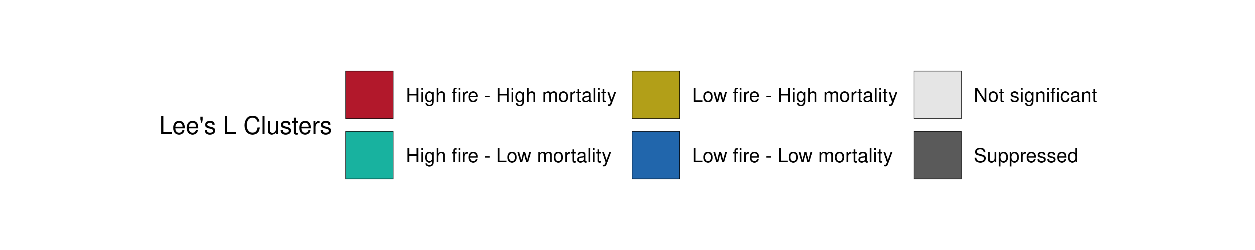 |

**Figure S-8** Bivariate associations between county-level burned area in a county-to-area county ratio (the total burned area from 1997-2003 in a county to total county area) and age-adjusted LCM rates (2016-2020) among females, accounting for the county-level prevalence of current smoking. Not significant denoted as *p* > 0.05.

| 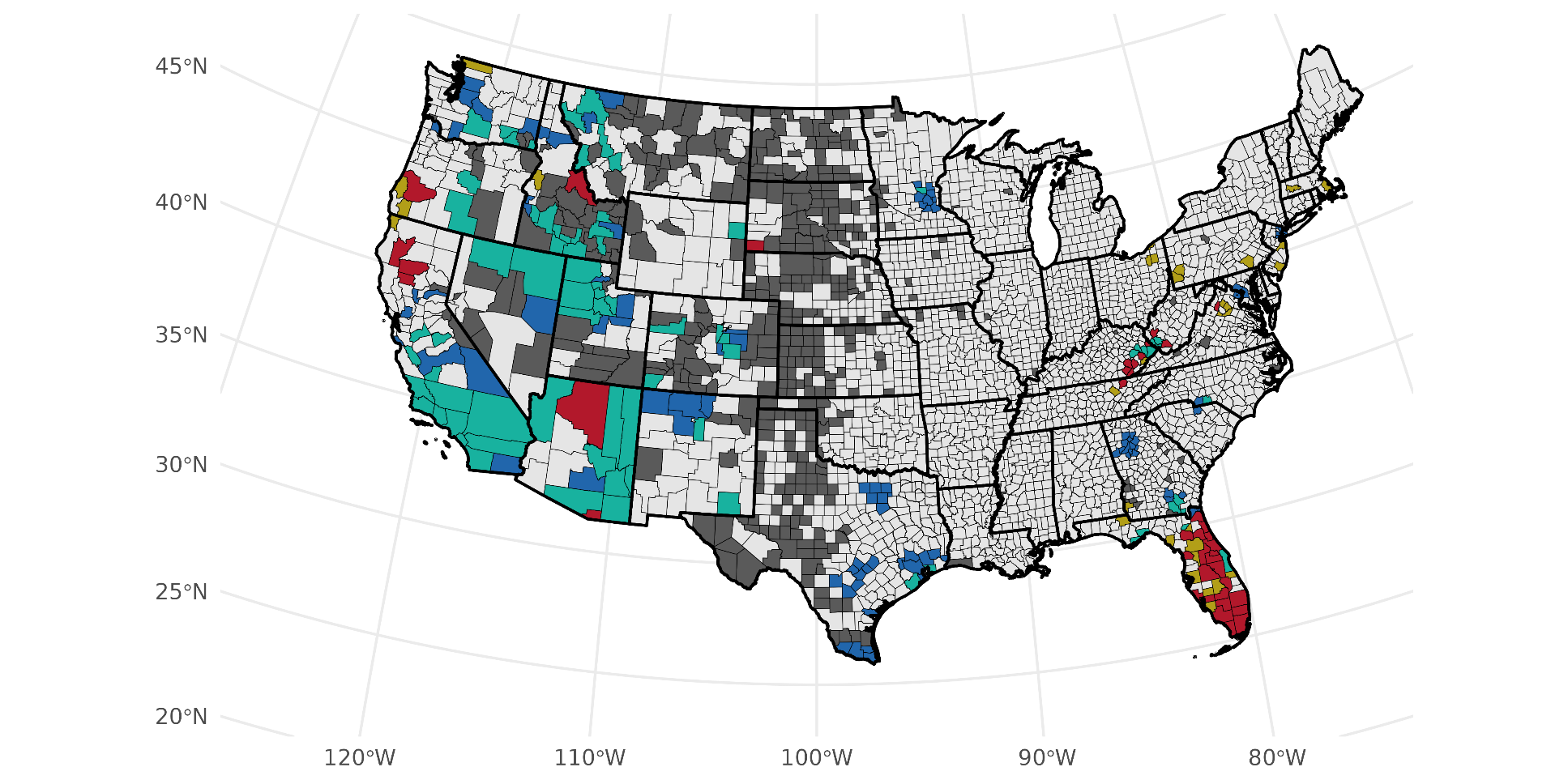 |
| --- |
| 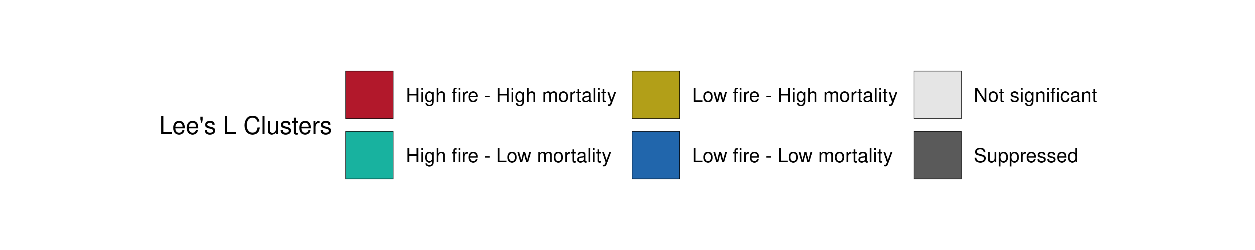 |

**Figure S-9** Bivariate associations between county-level burned area in a county-to-area county ratio (the total burned area from 1997-2003 in a county to total county area) and age-adjusted LCM rates (2016-2020) among males, accounting for the county-level prevalence of current smoking. Not significant denoted as *p* > 0.05.

| 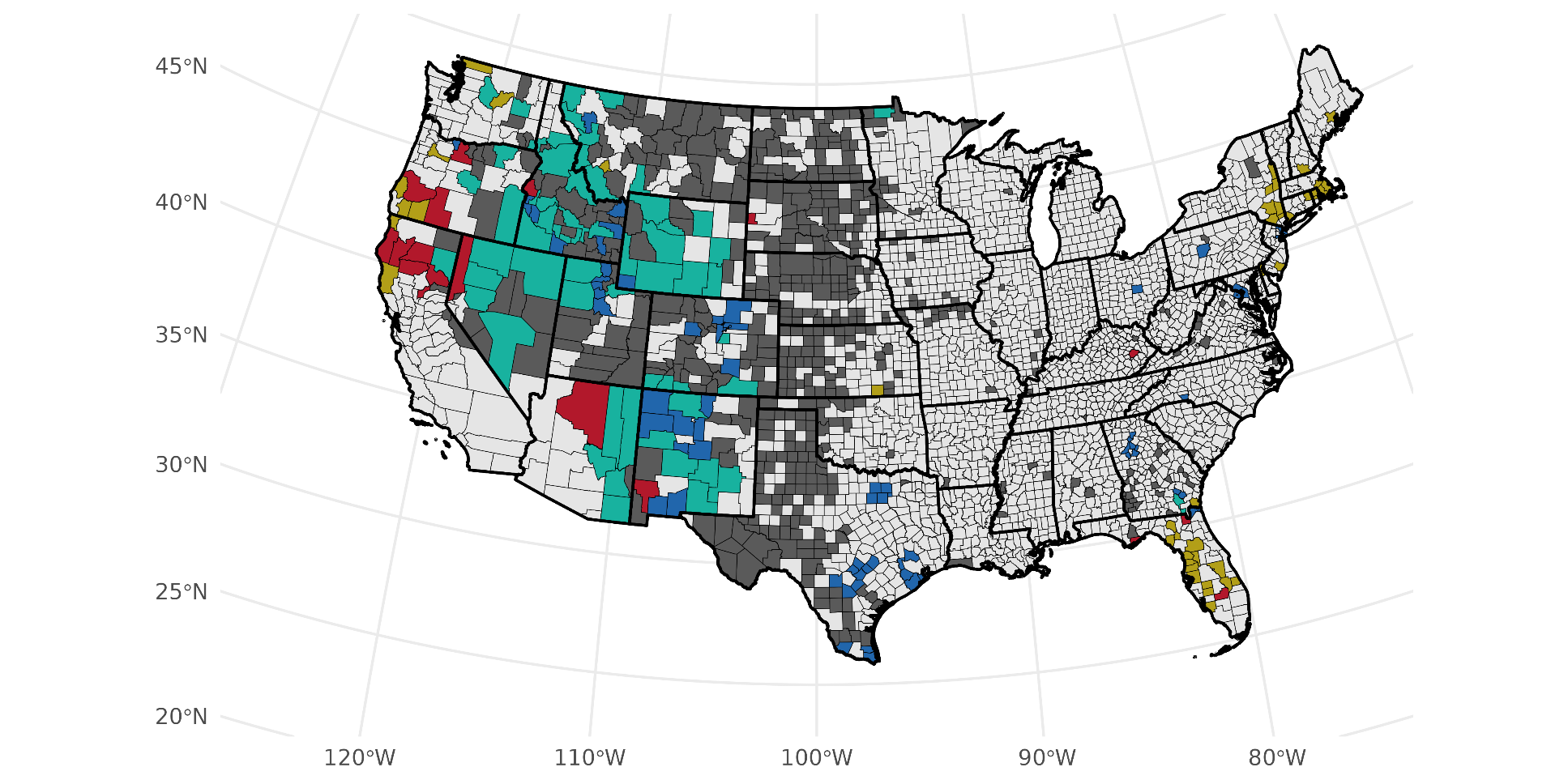 |
| --- |
| 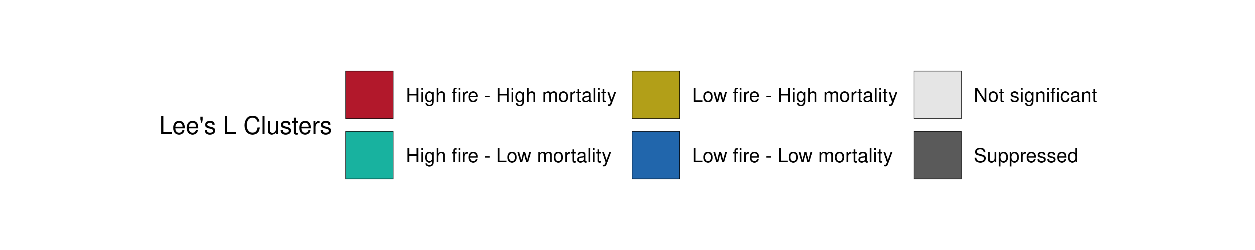 |

**Figure S-10** Bivariate associations between county-level burned area per 100,000 persons based on county population and age-adjusted LCM rates (2016-2020) among females, accounting for the county-level prevalence of current smoking. Not significant denoted as *p* > 0.05.

| 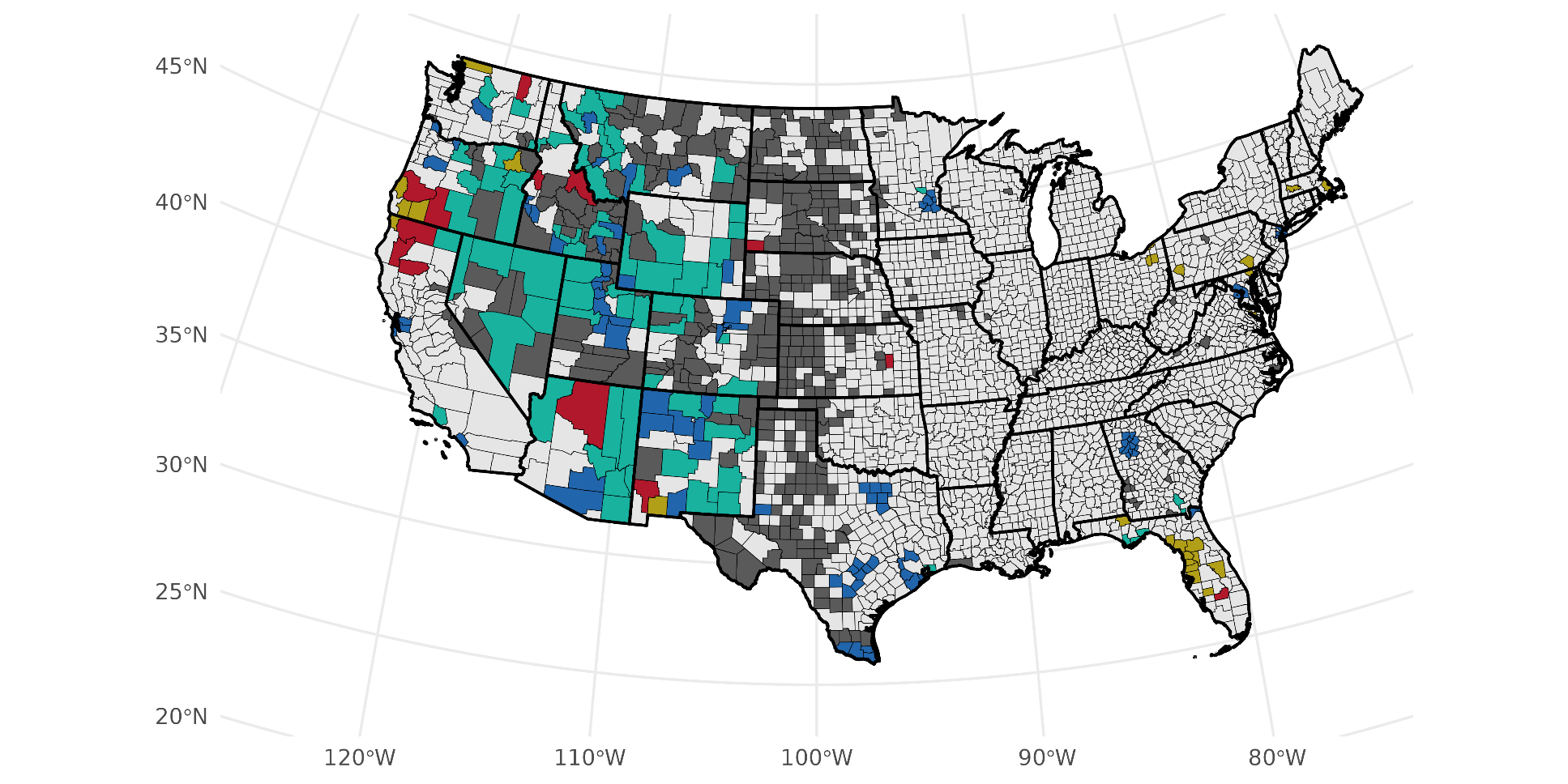 |
| --- |
| 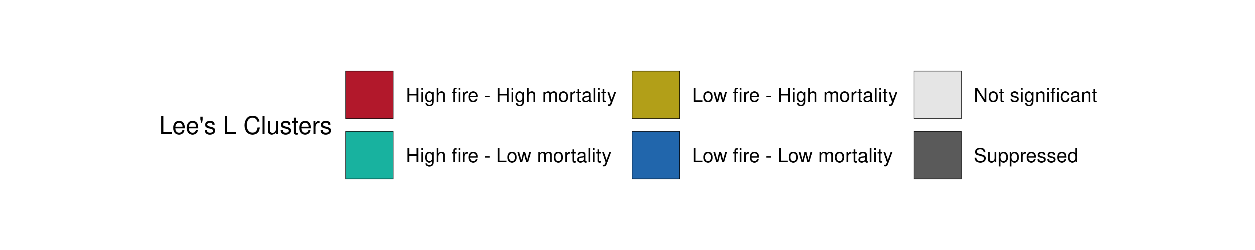 |

**Figure S-11** Bivariate between county-level burned area per 100,000 persons based on county population and age-adjusted LCM rates (2016-2020) among males, accounting for the county-level prevalence of current smoking. Not significant denoted as *p* > 0.05.

|  | Ever smokers | Current smokers |
| --- | --- | --- |
| Female | *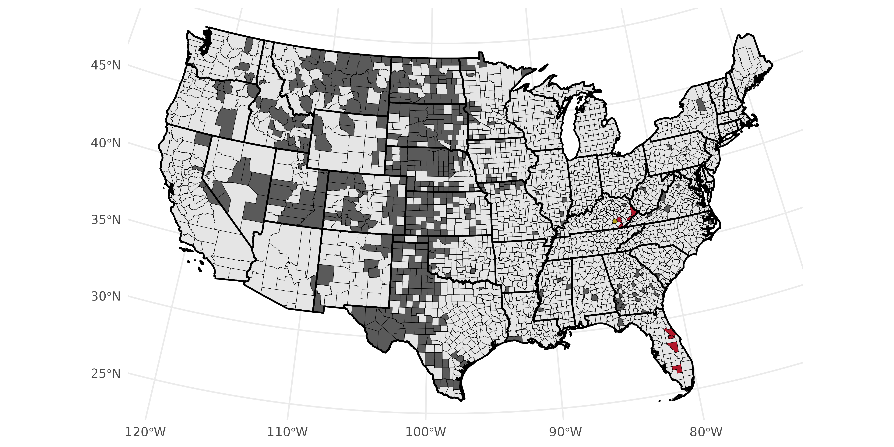* | *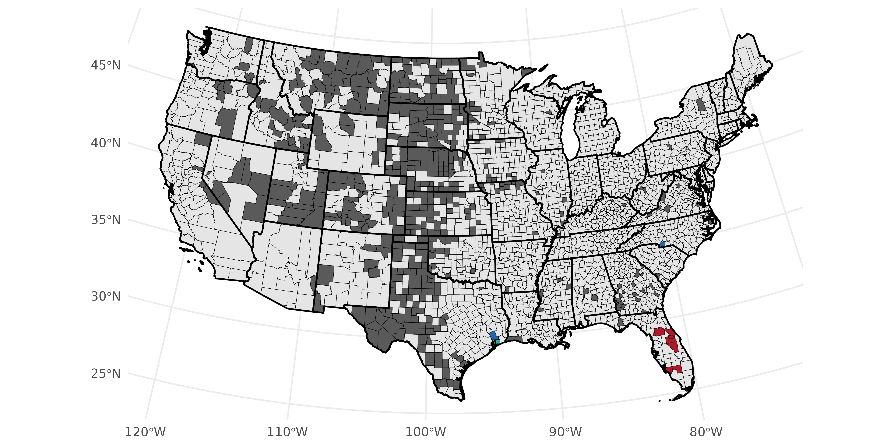* |
| Male | *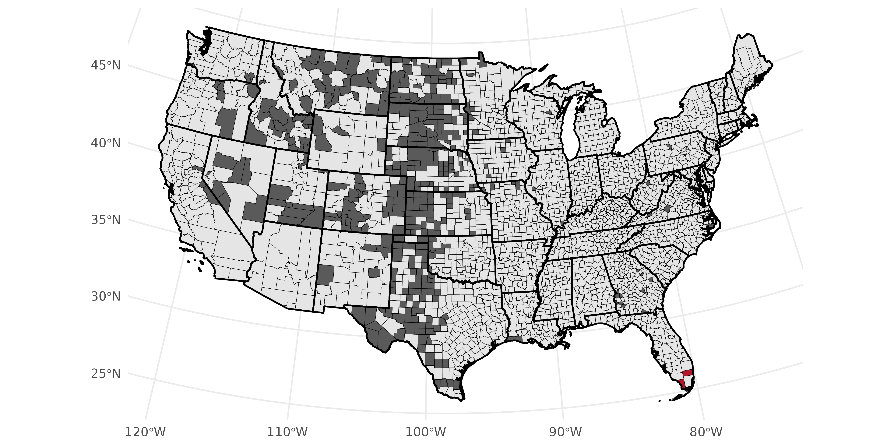* | *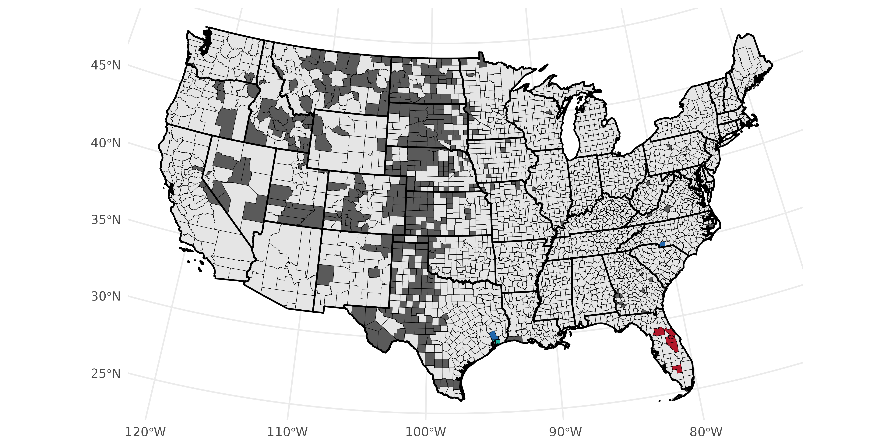* |


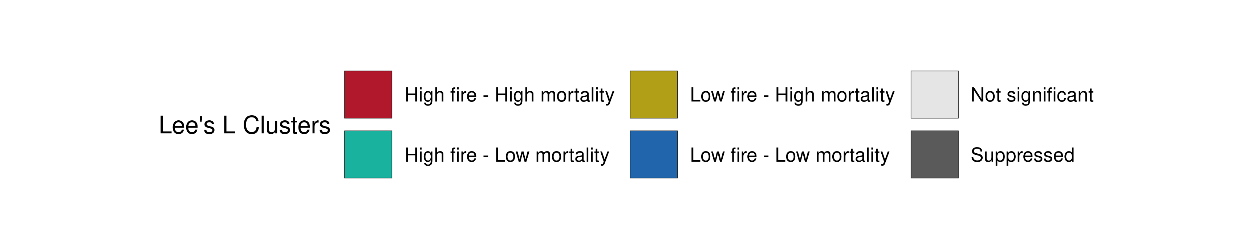


**Figure S-12** Bivariate associations with multiple comparison corrections between wildland fire density (# events per 1,000 km2 from 1997-2003 in a county), age-adjusted sex-specific (rows) LCM rates (2016-2020) and adjusted by significant county-level smoking prevalence status (columns). Detection is based on an alpha threshold of 0.05.

|  | Ever smokers | Current smokers |
| --- | --- | --- |
| Female | *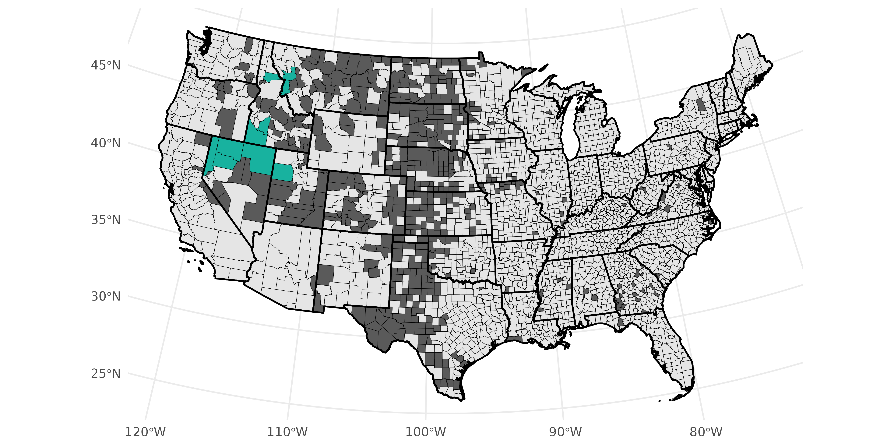* | *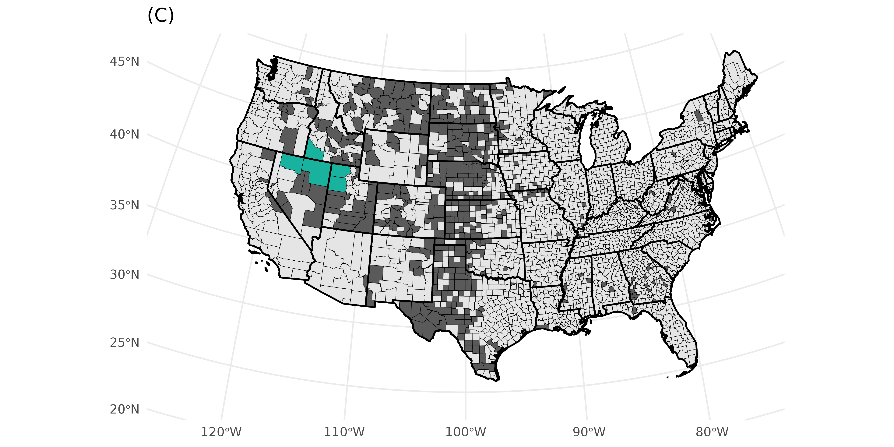* |
| Male | *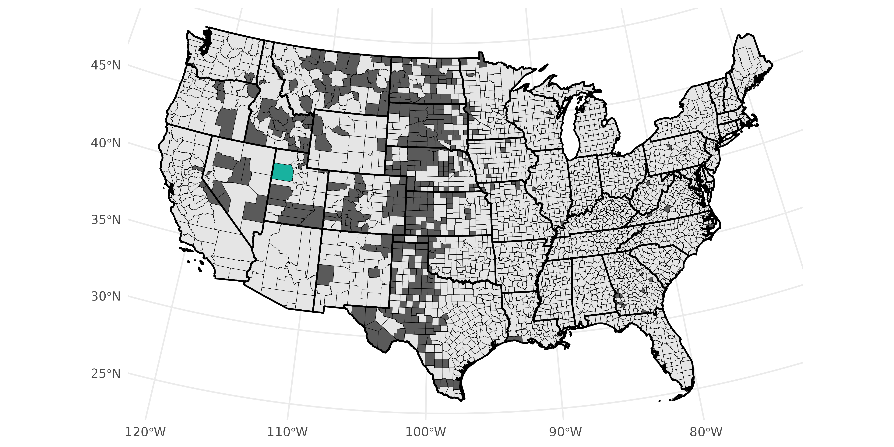* | *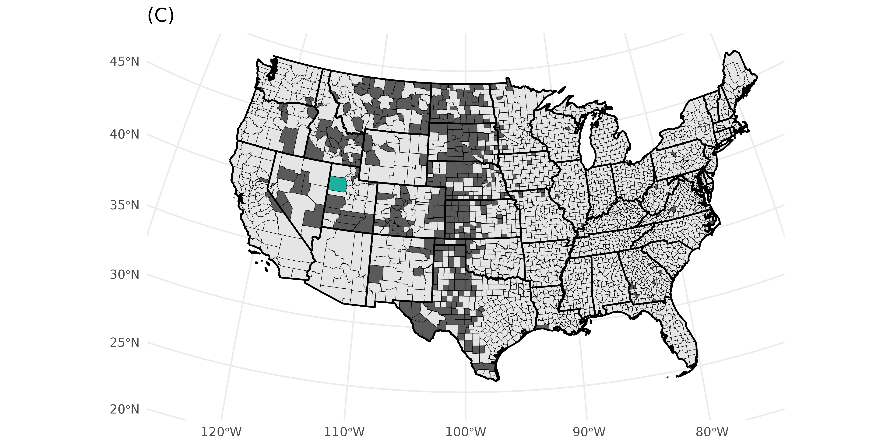* |


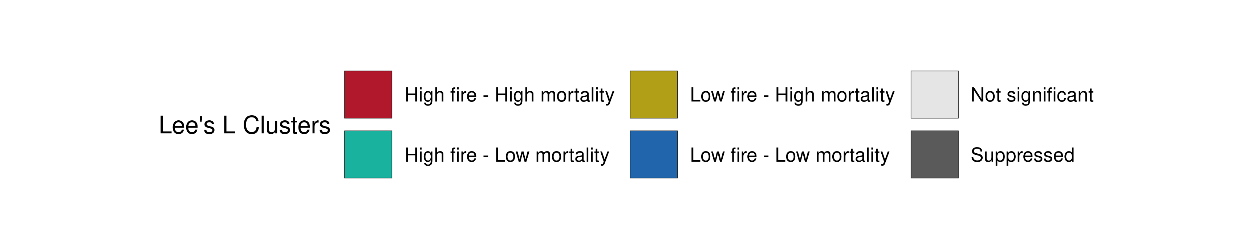


**Figure S-13** Bivariate associations with multiple comparison corrections between population-normalized county-level wildland fire density (# events per 100,000 persons, 1997-2003), age-adjusted sex-specific (rows) LCM rates (2016-2020) and restricted by significant county-level smoking prevalence status (columns). Detection is based on an alpha threshold of 0.05.

|  | Ever smokers | Current smokers |
| --- | --- | --- |
| Female | *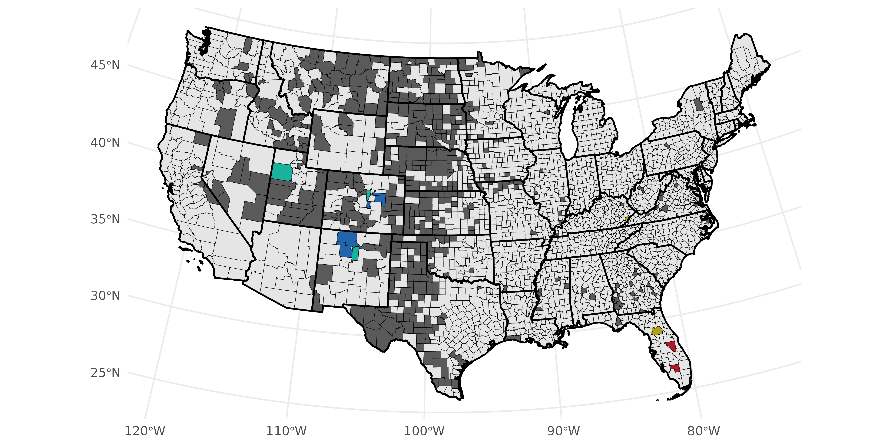* | *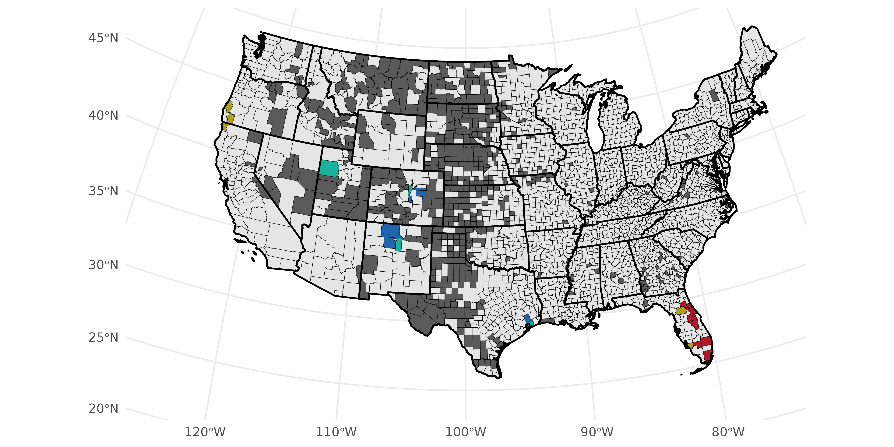* |
| Male | *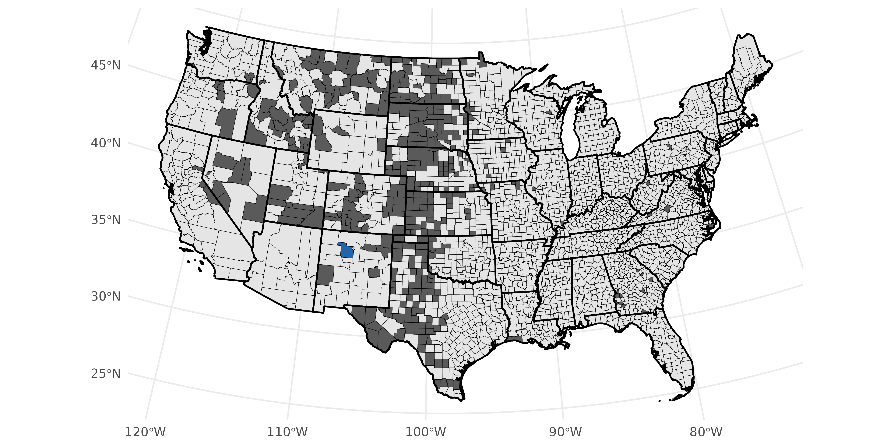* | *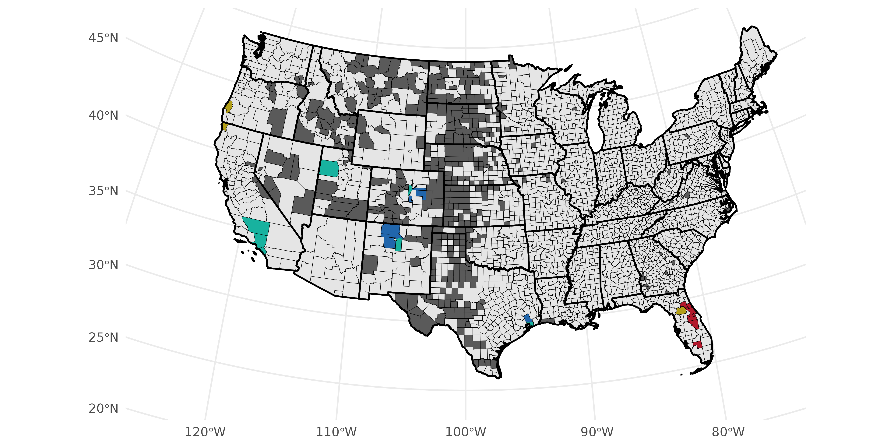* |


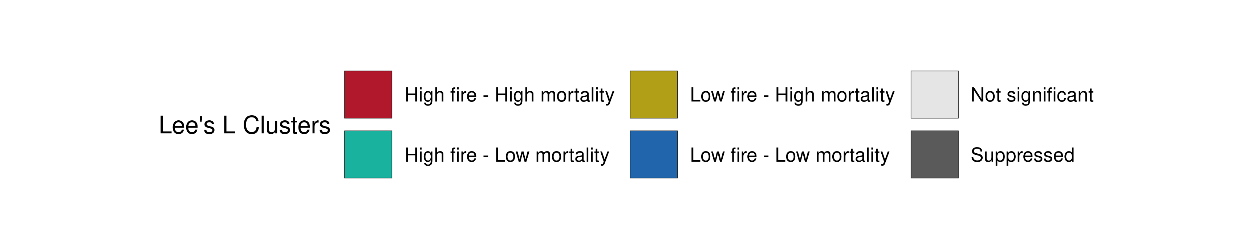


**Figure S-14** Bivariate associations with multiple comparison correction between wildland burned area in a county to area county ratio (total burned area from 1997-2003 in a county to total county area), age-adjusted sex-specific (rows) LCM rates (2016-2020) and restricted by significant county-level smoking prevalence status (columns). Detection is based on an alpha threshold of 0.05.


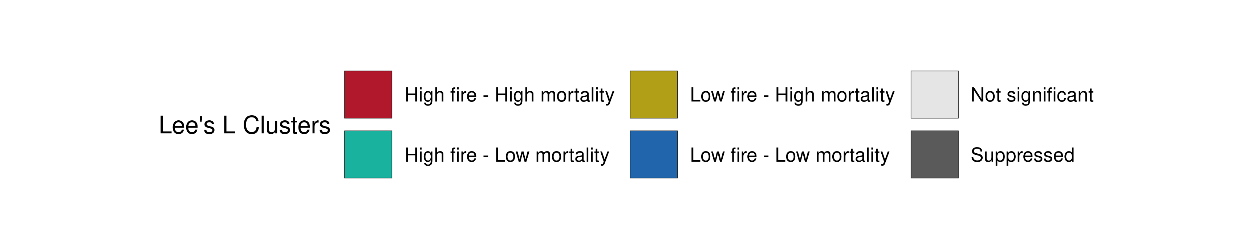


|  | Ever smokers | Current smokers |
| --- | --- | --- |
| Female | *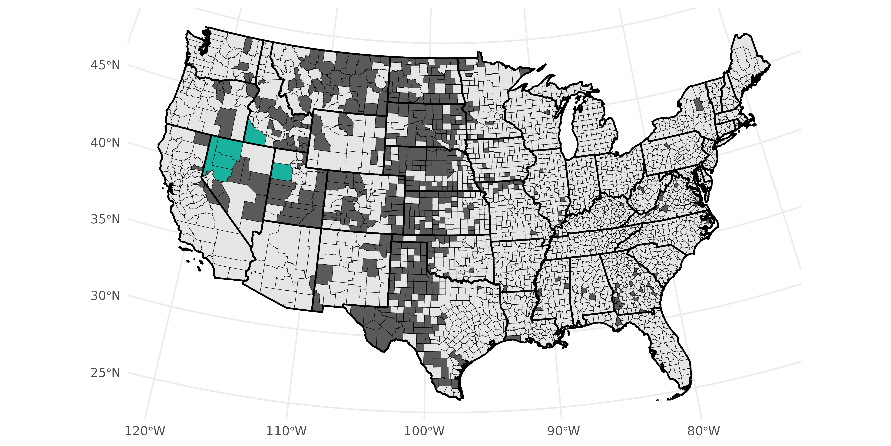* | *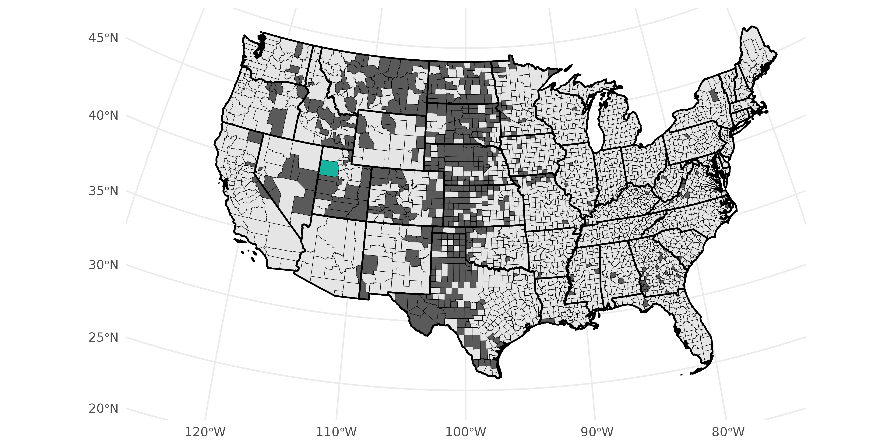* |
| Male | *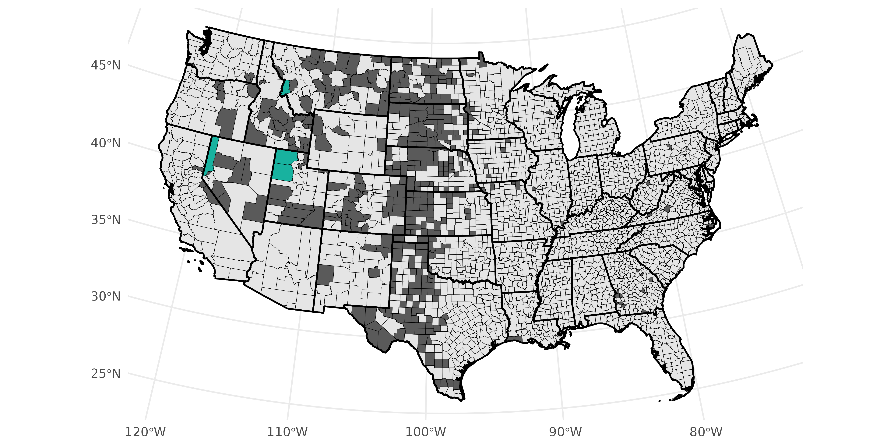* | *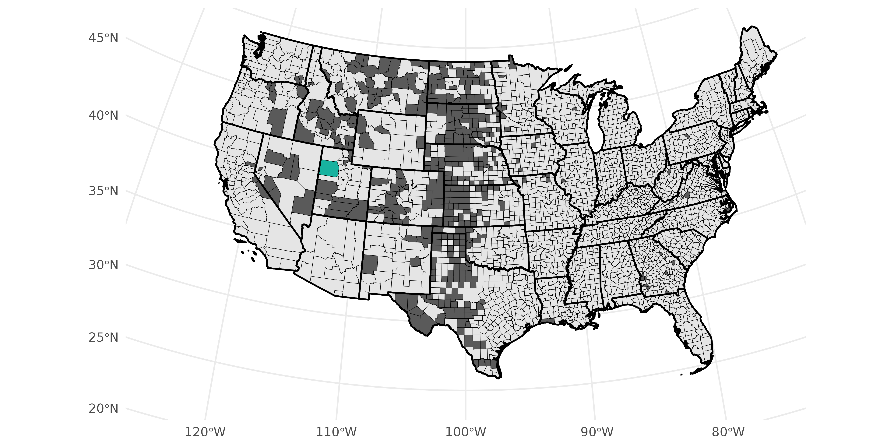* |

**Figure S-15** Bivariate associations with multiple comparison correction between county-level burned area per 100,000 persons based on county population, age-adjusted sex-specific (rows) LCM rates (2016-2020) and restricted by significant county-level smoking prevalence status (columns). Detection is based on an alpha threshold of 0.05.
